# Learning dispatching rules via novel genetic programming with feature selection in energy-aware dynamic job-shop scheduling

**DOI:** 10.1038/s41598-023-34951-w

**Published:** 2023-05-26

**Authors:** Adilanmu Sitahong, Yiping Yuan, Ming Li, Junyan Ma, Zhiyong Ba, Yongxin Lu

**Affiliations:** School of Mechanical Engineering, Xinjiang University, Urumqi, 830047 China

**Keywords:** Mechanical engineering, Computer science

## Abstract

The incorporation of energy conservation measures into production efficiency is widely recognized as a crucial aspect of contemporary industry. This study aims to develop interpretable and high-quality dispatching rules for energy-aware dynamic job shop scheduling (EDJSS). In comparison to the traditional modeling methods, this paper proposes a novel genetic programming with online feature selection mechanism to learn dispatching rules automatically. The idea of the novel GP method is to achieve a progressive transition from exploration to exploitation by relating the level of population diversity to the stopping criteria and elapsed duration. We hypothesize that diverse and promising individuals obtained from the novel GP method can guide the feature selection to design competitive rules. The proposed approach is compared with three GP-based algorithms and 20 benchmark rules in the different job shop conditions and scheduling objectives considered energy consumption. Experiments show that the proposed approach greatly outperforms the compared methods in generating more interpretable and effective rules. Overall, the average improvement over the best-evolved rules by the other three GP-based algorithms is 12.67%, 15.38%, and 11.59% in the meakspan with energy consumption (EMS), mean weighted tardiness with energy consumption (EMWT), and mean flow time with energy consumption (EMFT) scenarios, respectively.

## Introduction

Industrial world is confronted with numerous challenges. While production criteria have traditionally been a significant factor in the decision-making process, contemporary global circumstances necessitate a heightened focus on environmental concerns. An increase in population growth, coupled with an upsurge in consumption rates and a decline in energy reserves, poses a significant threat of an impending energy crisis on a global scale^1^. Moreover, energy consumption is consistently accompanied by pollution, posing a significant threat to human well-being. The manufacturing sector is frequently identified as the primary consumer of energy and generator of environmental pollution. Therefore, manufacturing businesses must seek effective strategies to reduce energy usage and carbon pollution in their production processes. Enhancing energy efficiency through upgrading production equipment is a viable option. However, the challenges of long research cycles and expensive investment costs present formidable obstacles that are not easily surmounted^2^. Several studies have demonstrated that energy consumption can be effectively reduced through the implementation of green scheduling, which involves the consideration of traditional scheduling objectives and environmental factors^3,4^. In sustainable manufacturing, the energy issue is taken into account at three levels: the product level, the machine level, and the production system level^5^. The present study centers on the production system level, with the aim of minimizing energy consumption without necessitating the re-engineering of machines or products.

The Job Shop Scheduling (JSS) problem has garnered significant attention from both academic and industrial circles due to its extensive practical implications in the fields of cloud computing and manufacturing. In practical scenarios, processes exhibit greater dynamism and susceptibility to interruptions, including but not limited to rush orders, cancellations, alterations in lot sizes, and machinery failures. The academic literature on scheduling has put forth various traditional optimization techniques such as dynamic programming^6^, branch-and-bound^7^, and meta-heuristics^8^ to address the dynamic job shop scheduling (DJSS) problem. These approaches cannot deal with unforeseen disruptions. In light of the mounting environmental pollution and energy conservation challenges confronting the manufacturing industry, it is imperative to contemplate the sustainability and energy utilization aspects of the manufacturing process. There is a limited body of literature that addresses the issue of dynamic energy-aware shop scheduling problems. Most existing studies have employed complete rescheduling techniques, which may pose a risk of instability^9,10^. In addition, it is worth noting that scheduling issues in dynamic scenarios are considerably more complex than those in static scenarios. Furthermore, the time required to obtain an optimal or even a high-quality solution is substantial. Therefore, it is necessary to design product scheduling so that they can react immediately to any potential deviations.

The utilization of dispatching rules has demonstrated to be a promising heuristic strategy owing to its adaptable nature, low temporal complexity, and rapid response to dynamic conditions^11^. In each decision situation, the dispatching rule decides the job with the highest priority value to be scheduled next when a machine is free. Numerous artificial dispatching rules have been developed for diverse workshop environments thus far. The complex interconnections among diverse waiting procedures and job shop conditions make it challenging, if not unfeasible, to manually recognize all the underlying associations for constructing an effective dispatching rule. Due to the insufficient performance of human-made dispatching rules, certain scholars are endeavoring to devise a hyper-heuristic approach that can identify and acknowledge flexible rules for effectively addressing the challenges associated with Job Shop Scheduling^12,13^.

Genetic Programming (GP) has been effectively used as a data-driven strategy to learn complicated and effective dispatching rules for complex manufacturing situations^14,15^. When compared to other hyper-heuristics methods, GP has the advantages of flexible encoding representation, a powerful searching engine, and applicability to a wide range of real-world applications. In recent decades, there has been a growing interest in utilizing GP-based heuristic methods to address scheduling issues in manufacturing processes. However, the sophisticated issue as energy aware shop scheduling in dynamic scenarios was seldom considered. On the other side, there have been substantial advancement in generating dispatching rules using GP methods for production scheduling. But most of these approaches prioritize algorithmic efficiency and generate rules that are excessively lengthy. Moreover, the efficacy of Genetic Programming in producing superior rules depends on the precise collection of terminal sets involving the most important job, machine, and job shop information. As the number of functions and terminals grows, the search space grows exponentially^16^. Preventing this exponential expansion of designed rules over generations is crucial for a number of reasons. First, the final tree offers benefits in terms of decreased computational cost, improved generalization, and simpler structure analysis. Second, simpler dispatching rules in compact mathematical structures are easier to understand the behavior compared with larger rules. Third, in practice, shortening evolving rules boosts their chances of being applied in industry, since smaller rules are easier for decision-makers to understand and apply in real-world production contexts^17^. For the above reasons, a feature selection approach is used to condense the GP search space in this research.

The utilization of feature selection techniques in the field of machine learning presents a feasible resolution to this hard problem. The technique has demonstrated successful implementation across a range of applications, covering tasks such as classification^18^, clustering^19^, and regression^20^. The integration of the feature selection process with the tree-based GP technique enables directed exploration of potential regions within the search space by concentrating on the most significant terminals. To the best of our knowledge, the application of feature selection to multiple production schedule variants is mostly unexplored territory. There have been various drawbacks to these techniques, which are covered below:

- (1) A common metric used by most feature selection approaches is the frequency with which each terminal appears in the best-evolving rule, which is then used to determine the terminal’s relative importance. The key negative aspect of this approach is that it might provide biased results because of the existence of redundant features.
- (2) Feature selection methods reported in the literature usually adapt offline selection mechanisms or a selection checkpoint to obtain a set of selected features. In addition to requiring substantial time and coding effort, this offline selection method may lose some well-structured individuals evolved during the feature selection phase.
- (3) Current approaches, which largely focus on rule interpretability through feature selection processes, do not consider the improvement in solution quality of scheduling rules in various job shop contexts.
- (4) Existing feature selection methods in GP have been developed for traditional job shop scheduling such as job shop scheduling, flexible job shop scheduling, or dynamic job shop scheduling. Although this is reasonable since these job shop environments are common types in scheduling, we believe that developing a feature selection approach that combined with GP in energy-aware dynamic job shop scheduling (EDJSS) may lead to better results.

Given the above, we propose an integration approach that combines a novel GP method with feature selection mechanism to design interpretable and effective rules for EDJSS. The main contributions of this article can be summarized as follows:

- (1) Offer a three-stage GP framework-based online feature selection technique that incorporates information from both the chosen features and a set of diverse individuals in the feature selection process.
- (2) Provide an integration strategy that combines a novel GP algorithm with an online feature selection process to create concise and high-quality dispatching rules in EDJSS. The innovative GP technique, connected the amount of population variety with the stopping criteria and the elapsed time to progressively alter the searching area of the GP algorithm from exploration to exploitation.
- (3) Evaluate the effectiveness of the suggested method in comparison to three GP-based algorithms and twenty representative rules from the existing research, considering three different scenarios: makespan with energy consumption (EMS), with mean weighted tardiness with energy consumption (EMWT), and mean flowtime with energy consumption (EMFT).

The rest of the paper is organized as follow: In Sect. “Related work”, the literature review is presented. Section “Problem formulation” describes the problem and formulates the mathematical model. Section “Proposed methods” details the proposed approach. Section “Experimental design” provides the experimental design. Section “Results and discussion” provides the computational results and at the end there is conclusions and future recommendations.

## Related work

### Energy optimization of workshop scheduling

Growing expenses and environmental awareness have led to a growing tendency to reduce energy consumption in conventional industrial processes. The Job-Shop Scheduling (JSS) problem is a well-known production scheduling problem that has been extensively studied in the literature. It is commonly observed in various real-world production systems that follow the job-shop layout^21^. The categorization of energy optimization research in job shop scheduling is based on three distinct groups, namely, objective research optimization, machining process optimization, and application of comprehensive methods. For the objective research optimization group, Giglio et al.^22^ presented a mixed-integer programming model aimed at addressing an integrated lot sizing and energy-efficient job shop scheduling problem. Gong et al.^23^ formulated a mathematical model for multi-objective optimization based on the double flexible job-shop scheduling problem. The model takes into account various indicators such as processing time, green production, and human factors. Mokhtari et al.^24^ created a multi-objective optimization framework that includes three distinct objectives, namely, the total completion time, the overall system availability, and the combined energy cost of production and maintenance operations in the context of flexible job shop scheduling. Yin et al.^25^ introduced a novel mathematical scheduling model with low-carbon emissions for the flexible job-shop setting, which aims to maximize productivity while minimizing energy consumption and noise pollution. For the machining process optimization, an energy consumption model was put forth by Wu et al.^26^ to calculate the energy cost for a machine in various states. L. Zhang et al.^27^ have developed a new mixed-integer linear mathematical model with the aim of optimizing machine selection, job sequencing, and machine on–off decision making for enhanced efficiency. Xu et al.^28^ introduced a feedback control approach for addressing the production scheduling problem in the context of the comprehensive method application. This method takes into account both energy consumption and makespan. Y. Zhang et al.^29^, have proposed a novel approach to real-time multi-objective flexible job shop scheduling. Specifically, they have developed a dynamic game theory-based two-layer scheduling method aimed at minimizing makespan, total workload of the machines, and energy consumption. In a speed scaling framework, Zhang et al.^30^ suggested a multi objective genetic algorithm to reduce the overall weighted tardiness and overall energy consumption for JSSP. In one-word, numerous efforts have been made to link the efficiency of conventional production scheduling with the total energy cos. Nevertheless, the models employed in these studies are deterministic, with a fixed number of jobs. Given that unforeseen disruptions are a common occurrence in many real-world settings, it is clear that static scheduling is insufficient to meet the demands of such environments. Instead, a more dynamic and responsive approach is needed.

The body of literature related to dynamic scheduling has extensively explored a multitude of works that address the impact of newly arrived jobs on diverse manufacturing systems. Many attempts ignored the cost of energy in favor of efficiency improvements for conventional scheduling issues. Within dynamic scenarios, the two most frequently employed strategies are complete rescheduling and schedule repair. Tang et al.^31^ employed an enhanced particle accumulation optimization algorithm to address a dynamic flexible flow shop problem with the objective of minimizing both makespan and energy consumption. This study differs from previous work on energy problems that it takes into account dynamic factors such as the arrival of new jobs and machine failures, rather than focusing solely on static problems. The study titled “Dynamic Scheduling of Multi-Task for Hybrid Flow-Shop Based on Energy Consumption” was presented by Zeng et al.^32^. It is deemed significant due to its incorporation of a time window for machine idle time as a constraint and the inclusion of makespan and energy consumption as objective functions. Although complete rescheduling may offer optimal solutions, it has the potential to cause instability and disruption to process flows, resulting in significant production costs. Conversely, schedule repair methods involve making revisions solely to a portion of the originally established schedule in response to changes in the manufacturing environment.

In summary, research has been conducted on energy-efficient scheduling problems in dynamic scenarios. The empirical evidence suggests that implementing a schedule repair strategy is a more viable approach for managing a dynamic manufacturing system in practical settings. However, there are still several limitations that need to be considered. An example of this is obtaining an updated schedule in a timely manner, particularly for manufacturing applications of large scale.

### GP on the design of dispatching rules

**Figure 1:**
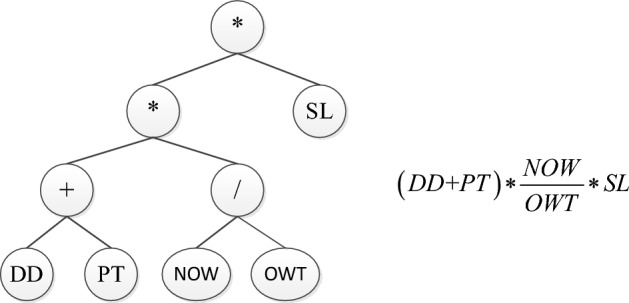
A GP individual tree example and its corresponding dispatching rule.

In contexts characterized by higher degrees of uncertainty and high load levels, dispatching rules are deemed more appropriate than predictive approaches. Over the course of recent decades, many different kinds of dispatching rules have been put forth in order to tackle various job shop scenarios and objective targets. The Genetic Programming (GP) approach serves as a hyper-heuristic method that enables the automatic design of novel dispatching rules through the manipulation of structural and parametric elements, without requiring extensive domain-specific knowledge. Furthermore, a comprehensive comprehension of the behavior exhibited by the evolved rules through the utilization of GP’s tree-based programs can be attained. Specifically, the GP algorithm produces individuals in the form of trees, utilizing a set of terminals (for leaf nodes) and a set of functions (for non-leaf nodes). Figure 1 gives an example of a parse tree and the corresponding dispatching rule. In this program, the terminal set consist of the variables $\left\{DD,PT,AT,OWT,SL\right\}$, and the function are composed of $\left\{+, *, \div \right\}$, where $\div$ is indicated by $/$. The priority of a job is calculated as $(DD+PT)*\frac{NOW}{OWT}*SL$, and is thus a linear combination of the processing time of the operation (PT), the due date of the job (DD), the current time (AT), the waiting time of the operation (OWT), and the slack time of the job (SL). The crossover operator is a stochastic process that involves the random selection of a sub-tree from each parent, followed by their exchange to generate two offspring. A sub-tree from the parent is chosen at random by the mutation operator, and it is then replaced with a new created sub-tree. The process for evaluating the tree starts with the application of the operator at the root node of the tree to the values acquired through the recursive evaluation of the left and right subtrees.

Burke et al.^11^ have presented a taxonomy of hyper-heuristic methodologies that is predicated on their search mechanisms, which encompass the selection and generation of hyper-heuristics. Furthermore, a succinct summary of hyper-heuristic implementations for diverse scheduling and combinatorial optimization problems was also furnished. According to the authors, GP is a suitable approach for devising dispatching rules in dynamic scenarios, despite its infrequent utilization for directly addressing production scheduling issues. Nguyen et al.^33^ have developed a unified framework for the automatic construction of dispatching rules using genetic programming. The authors presented an in-depth analysis of the fundamental elements and pragmatic concerns that must be taken into account prior to constructing a GP framework aimed at producing scheduling heuristics for production. That article has demonstrated a notable increase in the amount of research efforts related to the automated design of scheduling heuristics after 2010. Branke et al.^34^ gave a summary of relevant research on genetic programming hyper-heuristics techniques designed to solve production scheduling issues in the same setting. Furthermore, GP methodology has been employed to develop scheduling heuristics for production scheduling problems that exhibit a wide range of discriminative features. For instance, the GP approach has been applied to address the job arrivals and machine breakdowns in a dynamic environment^35^. Also, the GP technique has been utilized to tackle the dual-constrained flow shop scheduling problem that includes both machines and operators^36^.

In addition to the aforementioned research, numerous research endeavors have been undertaken with the objective of enhancing the efficacy of the GP methodology through different techniques. Park et al.^37^ enhanced the robustness of the GP approach for the DJSSP by utilizing ensemble learning and employing various combination strategies. Zhou et al.^38^ presented a novel approach for the dynamic flexible job shop problem (DFJSP) using a surrogate-assisted cooperative coevolution GP technique. The aforementioned approach was discovered to improve the computational efficacy and the offline acquisition of knowledge of the hyper-heuristic, all the while preserving its overall performance. However, sustainability issues are not taken into account in these papers. And there is a limited number of academic papers that utilize GP algorithms for learning dispatching rules in the context of energy-aware dynamic scheduling problems.

### Feature selection

The process of identifying the optimal feature set is widely recognized as a significant area of investigation within the domains of machine learning and data mining^39–41^. Feature selection can effectively decrease the search area, diminish data dimensionality, enhance interpretability of rules, and save time for training by carefully selecting important and noteworthy features. In EDJSS, a broad variety of workshop state characteristics (for example, the remaining time of each operation and the idle time of each machine) may be regarded as features to be included in the terminal set. It might be challenging to determine which features are beneficial for learning scheduling heuristics. Past research often included all possible features in the final terminal set. Thus, the designed scheduling heuristics have a wide variety of features, making them challenging to understand. In addition, a large set of terminals with redundant or irrelevant attributes creates an increasingly large and noisy search space, hence diminishing the GP's search power. Selecting key attributes for various scenarios is crucial in developing concise and understandable dispatching rules. The utilization of feature selection can serve as a viable approach to address the issue of job shop scheduling.

The feature selection techniques can be broadly classified into three categories, namely filter, wrapper, and embedding techniques^33^. For the most part, the following considerations prevent the aforementioned feature selection approaches from being directly applicable to job shop scheduling. First, in contrast to traditional machine learning occupations, the objective of job scheduling production is to assign priority to operations within a given waiting queue. Second, the acquisition of training data for job shop scheduling is limited to simulation models, whereas it is readily available in machine learning applications. Although GP has the capability to identify hidden associations among a subset of attributes, its efficacy and accuracy are limited. Previous investigations have indicated that even the most superior individuals possess certain features that are irrelevant and redundant. Stated differently, the capacity of GP is restricted.

Nonetheless, the feature ranking technique commonly employed, as suggested by Friedlander’s study^42^, exhibits a constraint whereby certain features are inaccurately assessed due to their occurrence in the redundant priority function. Therefore, Mei et al.^43^ introduced a new feature selection methodology in the context of GP. This approach evaluates the significance of features by assessing their contribution to the priority function. Despite its ability to more precisely identify significant features, this novel approach needs a substantial investment of computational resources to generate a diverse set of high-quality individuals. Subsequently, Mei et al.^44^ developed an enhanced feature selection algorithm that incorporates niching and surrogate techniques to generate superior dispatching rules within the context of DJSS. The study employed a niching-based genetic programming algorithm to initially generate a diverse set of high-quality individuals. Subsequently, an evaluation was conducted to determine the impact of the feature on the performance of high-quality rules, which was accomplished through the implementation of a weighted voting system. Ultimately, the chosen terminals were utilized to facilitate the development of optimal rules in subsequent runs of genetic programming. The methodology exhibits three significant limitations. First, this method continues to employ an offline feature selection mechanism, thereby incurring expensive computational costs. Second, even though the niching-based search framework has been shown to be more efficient in obtaining diverse rules compared to previous research, it requires further sophisticated experimentation to fine-tune the parameters of the niche. Third, despite the feature selection method being able to adequately identify relative feature subsets, the program size of the developed rules remains insufficiently compact. A unique two-stage GPHH architecture with feature selection and individual adaptive techniques was put out by Zhang et al.^45^ for the flexible DJSS problem in a later study. This method divides the entire GP procedure into two phases using a specified checkpoint. In the initial stage, a technique utilizing niching principles in conjunction with a surrogate module is employed to attain selected terminals. The replacement of the original terminal with the new one facilitates individual evolution after the generation of checkpoints during the second stage. The enhancement of rule performance was not taken into account, despite the fact that this feature selection framework was modified online and produced compact rules.

As previously mentioned, there is a growing body of academic research on the development of scheduling rules that incorporate feature selection or simplification. However, there remain gaps in the interpretability of the rules, and additional research is required to assess the influence of improved strategies on the effectiveness of the rules. Most importantly, few studies have considered both feature selection mechanism and rule quality improvement using GP algorithm for EDJSS. Therefore, this work aims at designing interpretable and high-quality rules simultaneously for the EDJSS problem. The study aims to showcase the efficacy of the obtained dispatching rules in producing high-quality schedules for the model problem in diverse experimental scenarios by means of computational experiments.

## Problem formulation

### Problem description

The Energy-Aware Dynamic Job Shop Scheduling (EDJSS) is a variant of the Job Shop Scheduling (JSS) problem that incorporates machine speed scaling. This approach allows for machines to operate at varying speed levels depending on the specific job being processed. A set of $n$ jobs should be processed on a group of $m$ machines in the shop. Each job follows a unique processing path, where the machines are utilized and their order of operation may vary. Each job is assumed to have a basic processing time. The fundamental processing time inside a task for each operation is predetermined. A machine cannot be fully powered down unless all its scheduled operations have been completed.

Current research on energy-efficient production scheduling focuses on control of machine ON/OFF, speed scaling, and time-of-use-based pricing of electricity^46^. The majority of current research on time-of-use power pricing and machine ON/OFF management bases its conclusions on the unreasonable assumption that machines' processing speeds are constant in today’s industrial environments. In certain manufacturing systems, the implementation of machine ON/OFF control may not be feasible due to the potential harm that may be inflicted upon the machines and the increased energy consumption associated with frequent restarts. The present paper employs a speed-scaling mechanism to aid in the modeling and coding process. Specifically, each machine is equipped with a finite and discrete speed set.

### Mathematical model

The following section introduces the energy-aware modelling for the production scheduling problem that occurs in a job shop floor considering dynamic job arrival. Table 1 presents the indexes, parameters, and variables utilized in the current model.

**Table 1:** Notations of mathematical formulation.

$i,h,{i}^{^{\prime}}$	Index of the jobs, $i=1,\cdots n$
$j, { j}^{^{\prime}}$	Index of the operations
$k,{k}^{^{\prime}}$	Index of the machines, $k=1,\cdots m$
$z$	Index of machine speed levels
${l}_{i}$	Total number of operations of job $i$,${l}_{i}\le m$
$L$	Total number of speed levels of each machine
${O}_{i,j}$	$j$ th operation of job $i$
${t}_{i,j}$	Processing time of operation ${O}_{i,j}$
${v}_{z}$	$z$ th speed of each machine
${PM}_{kz}$	Processing power of machine $k$ when its machine speed is set to ${v}_{z}$
${PS}_{k}$	Standby power of machine $k$
${PST}_{k}$	Setup power of machine $k$
${ST}_{ihk}$	Sequence-dependent setup time for processing job $h$ immediately after job $i$ on the same machine $k$
${ST}_{ohk}$	Setup time of job $h$ on machine $k$ when job $h$ is the first job processed on machine $k$
${D}_{i}$	Due date of the job $i$
$H$	Large enough integer
${C}_{i,j}$	Completion time of operation ${O}_{i,j}$
${C}_{i}$	Completion time of machine $k$
${C}_{max}$	Completion time of all jobs
${w}_{i}$	The weight of the job $i$
${f}_{i}$	The flowtime of job $j$
${\mathbb{T}}$	Set of delayed jobs
${\mathbb{C}}$	Set of completed jobs

Objective functions and constraints:

1 $$MS = \max \,\left\{ {C_{i} \,\left| {i\, = \,1,2, \cdots ,n} \right.} \right\},$$

2 $$MWT = {\mkern 1mu} {\mkern 1mu} \frac{{\sum\limits_{{i \in {\mathbb{T}}}} {w_{i} {\mkern 1mu} \left( {C_{i} {\mkern 1mu} - {\mkern 1mu} D_{i} } \right)} }}{{\left| {\mathbb{T}} \right|}},$$

3 $$MFT = \frac{{\sum\limits_{{i \in {\mathbb{C}}}} {f_{i} } }}{{\left| {\mathbb{C}} \right|}},$$

4 $$\begin{aligned} TEC & = E_{1} + E_{2} + E_{3} { = }\sum\limits_{i = 1}^{n} {\sum\limits_{j = 1}^{{l_{i} }} {\sum\limits_{k = 1}^{m} {\sum\limits_{z = 1}^{L} {z_{ijkz} \frac{{t_{ij} }}{{v_{z} }}} } } } PM_{kz} { + }\sum\limits_{k = 1}^{m} {\sum\limits_{i = 0,h = 1}^{n} {y_{ihk} ST_{ihk} PST_{k} } } \\ { + }\sum\limits_{k = 1}^{m} {\left[ {C^{k} PS_{k} - \sum\limits_{i = 1}^{n} {\sum\limits_{j = 1}^{{l_{i} }} {\sum\limits_{z = 1}^{L} {z_{ijkz} \frac{{t_{ij} }}{{v_{z} }}PS_{k} - \sum\limits_{i = 0,h = 1}^{n} {y_{ihk} ST_{ihk} PS_{k} } } } } } \right]} , \\ \end{aligned}$$

S.t.

5 $$C_{i^{\prime}j^{\prime}} + \frac{{t_{ij} }}{{v_{z} }}z_{ijkz} + ST_{i^{\prime}ik} y_{i^{\prime}ik} \le C_{ij} + \left( {1 - y_{i^{\prime}ik} } \right)H,$$

where $$i = 1,2, \cdots ,n$$; $$i^{\prime} = 0,1,2, \cdots ,n$$; $$i \ne i^{\prime}$$; $$j = 1,2, \cdots ,l_{i}$$; $$j^{\prime} = 1,2, \cdots ,l_{i}$$; $$z = 1,2, \cdots ,L$$.

6 $$C_{{i\left( {j - 1} \right)}} + \frac{{t_{ij} }}{{v_{z} }}z_{ijkz} + ST_{i^{\prime}ik} y_{i^{\prime}ik} \le C_{ij} + \left( {1 - \gamma_{i^{\prime}ik} } \right)H,$$

where $$i = 1,2, \cdots ,n$$; $$i^{\prime} = 0,1,2, \cdots ,n$$; $$i \ne i^{\prime}$$; $$k,k^{\prime} = 1,2, \cdots ,m$$; $$k \ne k^{\prime}$$; $$j = 2, \cdots ,l_{i}$$; $$z = 1,2, \cdots ,L$$.

7 $$\frac{{t_{ij} }}{{v_{z} }}z_{ijkz} + ST_{0ik} y_{0ik} \le C_{ij} + \left( {1 - y_{0ik} } \right)H,$$

where $$i = 1,2, \cdots ,n$$; $$i^{\prime} = 0,1,2, \cdots ,n$$; $$i \ne i^{\prime}$$; $$k,k^{\prime} = 1,2, \cdots ,m$$; $$k \ne k^{\prime}$$; $$j = 2, \cdots ,l_{i}$$; $$z = 1,2, \cdots ,L$$.

8 $$\sum\limits_{{i^{\prime} = 0}}^{n} {y_{i^{\prime}ik} } = 1,$$

where $$i = 1,2, \cdots ,n$$; $$i \ne i^{\prime}$$; $$k = 1,2, \cdots ,m$$.

9 $$\sum\limits_{i = 1}^{n} {y_{i^{\prime}ik} } = 1,$$

where $$i^{\prime} = 0,1, \cdots ,n$$; $$i \ne i^{\prime}$$; $$k = 1,2, \cdots ,m$$.

10 $$\sum\limits_{z = 1}^{L} {z_{ijkz} } = 1,$$

where $$i = 1,2, \cdots ,n$$; $$j = 1,2, \cdots ,l_{i}$$; $$k = 1,2, \cdots ,m$$.

Equation (1) denotes as the makespan, which refers to the maximum time taken for the completion of all jobs. Equation (2) represents the mean weighted tardiness of all jobs. Equation (3) is the mean flowtime of all jobs. Equation (4) represents the total energy consumption of all machines, which can be divided three distinct components: processing energy ${E}_{1}$, standby energy ${E}_{2}$, and setup energy ${E}_{3}$. Constraint (5) guarantees that an operation may be processed on a machine only after the proceeding operation and the required preparatory activity have been completed. Constraint (6) specifies the priority relationship for each job's activities, such that the completion time of one operation must be greater than that of the previous operation, taking into account processing and setup durations. Constraint (7) outlines the precedence relation among the operations of a given job, specifically when that job is the initial one that begins processing on the machine. According to Constraint (8), a job is required to have a single predecessor, apart from the first job on the machine. Constraint (9) means that when a job has finished processing on a machine, one and only one different job can be selected for processing next. According to Constraint (10), it is inferred that during the processing of an operation on a machine, only one speed setting can be chosen and it cannot be altered.

## Proposed methods

### Framework of proposed approach

Based on the Zhang’s research^45^, which states the information of both the selected features and investigated individuals during the feature selection process can make a good contribution on evolving interpretable rules. This paper proposes a novel GP algorithm with online feature selection to design interpretable and high-quality dispatching rules for EDJSS (The pseudo-code of the proposed algorithm, Supplementary Information). The proposed strategy comprises of two crucial components, as follows:

- (1) A three-stage GP framework is developed to extract information from selected features and promising individuals to evolve compact and interpretable rules.
- (2) A novel GP approach involving dynamic diversity management is proposed to enable a gradual transition from exploration to exploitation. This novel GP employs a replacement strategy that combines penalties based on distance-like functions with a multi-objective Pareto selection based on correctness and simplicity.

**Figure 2:**
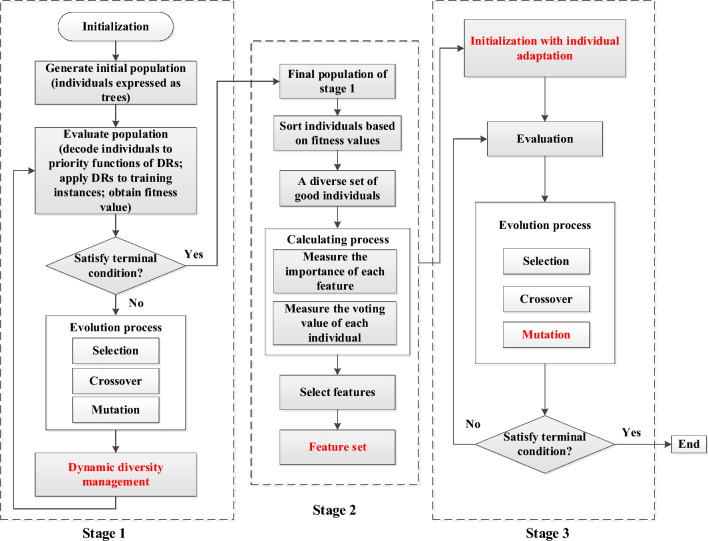
Flowchart of the proposed novel GP with online feature selection.

Figure 2 illustrates the suggested three-stage GP framework. In stage 1, a new general methodology for GP is presented that considers dynamic diversity management, considering the stopping criterion and elapsed time, with the aim of obtaining a diverse set of good individuals for feature selection. A key component of this diversity management strategy is the dynamic penalty system, which accounts for the degree of similarity between individuals in the replacement phase. In this manner, a diversified collection of optimal dispatching rules can be constructed, which is essential for achieving high precision in feature selection. The feature selection approach is used in stage 2 to extract feature subsets for the various job shop scenarios based on the final population obtained in stage 1. At stage 3, the traditional GP approach is utilized to create more concise and high-quality rules based on the final population with individual adaptation and the terminals that have been chosen. It is worth noting that the initialization and mutation procedure is different from the standard GP. In the initialization procedure, the final population of stage 1 is employed as the initial population. Constructing trees incorporating only a random subset of the features, which in turn avoids redundant branches.

### A novel GP method

This novel GP presents a proposal that hypothesizes that incorporating diversity management into the stopping criterion and the elapsed time for execution could yield further advantages in the GP domain. A unique replacement phase was designed using this principle. The inclusion of a penalty in the replacement phase to prevent the survival of too similar individuals is one of the most crucial aspects of this replacement strategy. The concept of similarity is established on distance-related functions that are dependent on the problem, while the concept of excessive similarity is dynamic. A threshold distance is established initially to differentiate between penalized and unpenalized individuals. Subsequently, the threshold undergoes a linear reduction throughout the course of the evolutionary process, ultimately reaching a value of 0 upon completion of the optimization procedure. The application of a penalty function serves to enhance the survival of a range of solutions that may be less optimal but exhibit greater diversity. Simultaneously, the utilization of a dynamic threshold mechanism serves to concentrate the search efforts towards the most promising regions during the final stages of the optimization process.

#### The replacement strategy

Algorithm 1 describes the pseudo-code of the suggested replacement approach in the GEP algorithm. The goal of the algorithm is to select the required number of survivors $n$ to create a new population ${P}_{new}$ for the following generation. Originally, a group of candidates $C$ is created by adding the current population $P$ and offspring $O$. As the fitness in this study is to be decreased, the candidates with the lowest values are selected, eliminated from the pool of candidates, and employed to establish new populations. The candidates are then penalized further by calculating a threshold $(D)$ value (line 4). Following the above initial procedures, $n-1$ iterations are used to choose survivors from the candidate set to form the new population (see lines 5–15). At each iteration, the algorithm divides the candidates into the penalized set (${C}_{p}$) and the on-penalized set (${C}_{np}$) (line 6). To be more explicit, every candidate whose distance to the closest survivor is less than the threshold $D$ is categorized as either a penalty candidate or a non-penalized candidate. In the situation that there are candidates that have not been punished, a multi-objective strategy that takes into account aspects such as fitness and simplicity is used to choose randomly dominated candidates, while penalized candidates are ignored (lines 7–9). Conversely, if all candidates are penalized, the algorithm selects the candidate with the greatest distance (line 11). Under these circumstances, it may suggest that population variety is too constrained, therefore choosing the individual which is the farthest away appears more promising. The chosen candidate is then dropped from the list of candidates list and added to the new population.
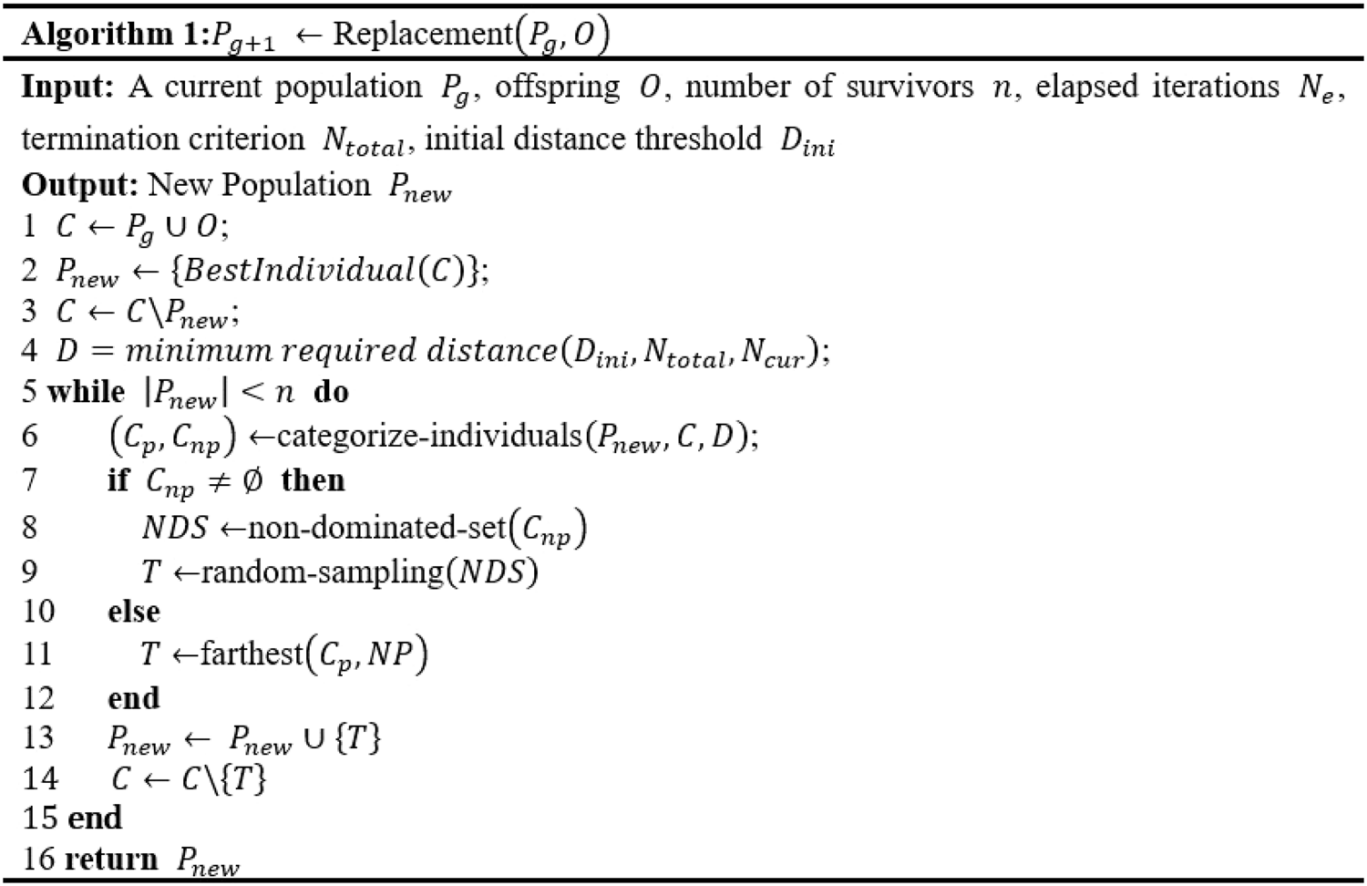


#### Phenotypic characterization of dispatching rules

The threshold in Algorithm 1 is determined by the minimum required distance function, which is then used to discriminate between those who are penalized and those who are not. Throughout the course of evolution, the threshold value lowers linearly. This suggests that the acceptance of increasingly related individuals occurs with each cycle, shifting the emphasis from exploration to exploitation. Thus, in the last step of optimization, the dynamic threshold automatically leads the search in the direction of the most promising regions. The value of threshold $D$ is determined in this study as follows:

11 $$D={D}_{ini}-\frac{{D}_{ini}\times {N}_{e}}{{N}_{total}},$$

12 $${D}_{ini}=0.5\times {DIS}_{ave(P)},$$

13 $${DIS}_{ave}\left(P\right)=\frac{1}{\left|P\right|}{\sum }_{i=1}^{\left|P\right|}DIS\left(P\left({r}_{i}\right),P\backslash P({r}_{i})\right),$$

where ${D}_{ini}$ is the initial distance value, ${DIS}_{ave(P)}$ is the average of the closest distance between individuals in a population $P$, ${N}_{e}$ is the elapsed iterations, ${N}_{total}$ is the stopping criterion.

The utilization of a distance measure, $DIS({r}_{1},{r}_{2})$ is required in the aforementioned threshold function to determine the distance between rules ${r}_{1}$ and ${r}_{2}$. In contrast to conventional tree distance metrics such as $ed2$ distance, it is feasible for two rules with distinct genotype structures to arrive at identical behavioral decisions in GP tree. Therefore, the distance measure should take into account differences in phenotypic behavior rather than genetic structure. The phenotypic characterization of dispatching rules used in this study is based on decision vector^47^, a list of all decisions taken by $r$ in a given set of decision scenarios ${\varvec{\Omega}}$. To get the ranking vector ${k}_{ref}$, all candidate tasks are first ranked using the reference dispatching rules ${r}_{ref}$. In order to produce the ranking vector ${k}_{r}$, the rule $r$ to be characterized is also applied to rank the tasks. Then, the next step is to get the index $j$ of the tasks that have the greatest priority as determined by the reference rule ${r}_{ref}$. Finally, the $i$ th element of the characteristic vector is assigned the rank of the $j$ th job in the ranking vector. The phenotypic characterization of rules is provided in pseudocode in Algorithm 2.
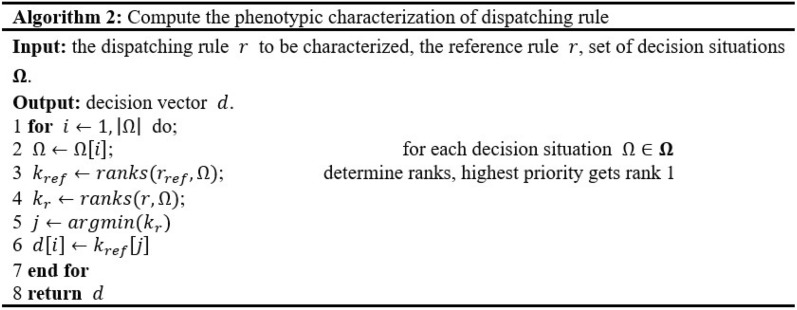


### Feature selection

This study adopts the feature selection concept in Mei’s research^43^, which posits that the significance of features is determined by their relevance to individual fitness and their contribution to individuals. Thus, we propose a feature selection mechanism involves three main steps. First, a diverse set of good individuals $\widetilde{R}$ are selected from the final population at stage 1 based on the fitness value. Second, the contribution of each feature to the fitness of an individual in $\widetilde{R}$ is evaluated, and if a feature contributes to that fitness, an individual in $\widetilde{R}$ will vote for it. Ultimately, the feature is chosen if more individuals vote for it than against it. The pseudo code for the feature selection method is shown in Algorithm 3.

#### Contribution of features

The contribution of each feature $f$ to an individual $\widetilde{r}$ is quantified by Eq. (14), where $fitness(\widetilde{r}\left|f=1)\right.$ represents the fitness value of the rule $\widetilde{r}$ that set the feature $f$ with the constant of 1. For examples, $(PT+WINQ\left|PT=1)\right.=1+WINQ$. A positive value of $Con\left(f,\widetilde{r}\right)$ indicates that eliminating the feature $f$ has a negative impact on the rule's performance. Thus, the rule $\widetilde{r}$ may provide voting weight to the measured feature.

14 $$Con\left(f,\widetilde{r}\right)=fitness(\widetilde{r}\left|f=1)\right.-fitness\left(\widetilde{r}\right).$$

#### Feature selection decision

As the goal functions provided in this work are intended to be reduced, dispatching rules with lower fitness have higher voting weights. Therefore, a dispatching rule’s “voting weight” should be a monotonically decreasing function of its fitness. Equations (15), (16), (17) and (18) describes the calculation. Ultimately, the feature $f$ is selected if the weight voting for it is larger than the weight voting against it (see lines 10 to 13).

15 $$w\left(r\right)=max\left\{\frac{u\left(r\right)-{u}_{min}}{{u}_{max}-{u}_{min}},0\right\},$$

16 $$u\left(r\right)=\frac{1}{1+fitness(r)},$$

17 $${u}_{max}=\frac{1}{1+min\left(fitness(r)\left|r\in R\right.\right)},$$

18 $${u}_{min}=\frac{1}{1+max\left(fitness(r)\left|r\in R\right.\right)}.$$

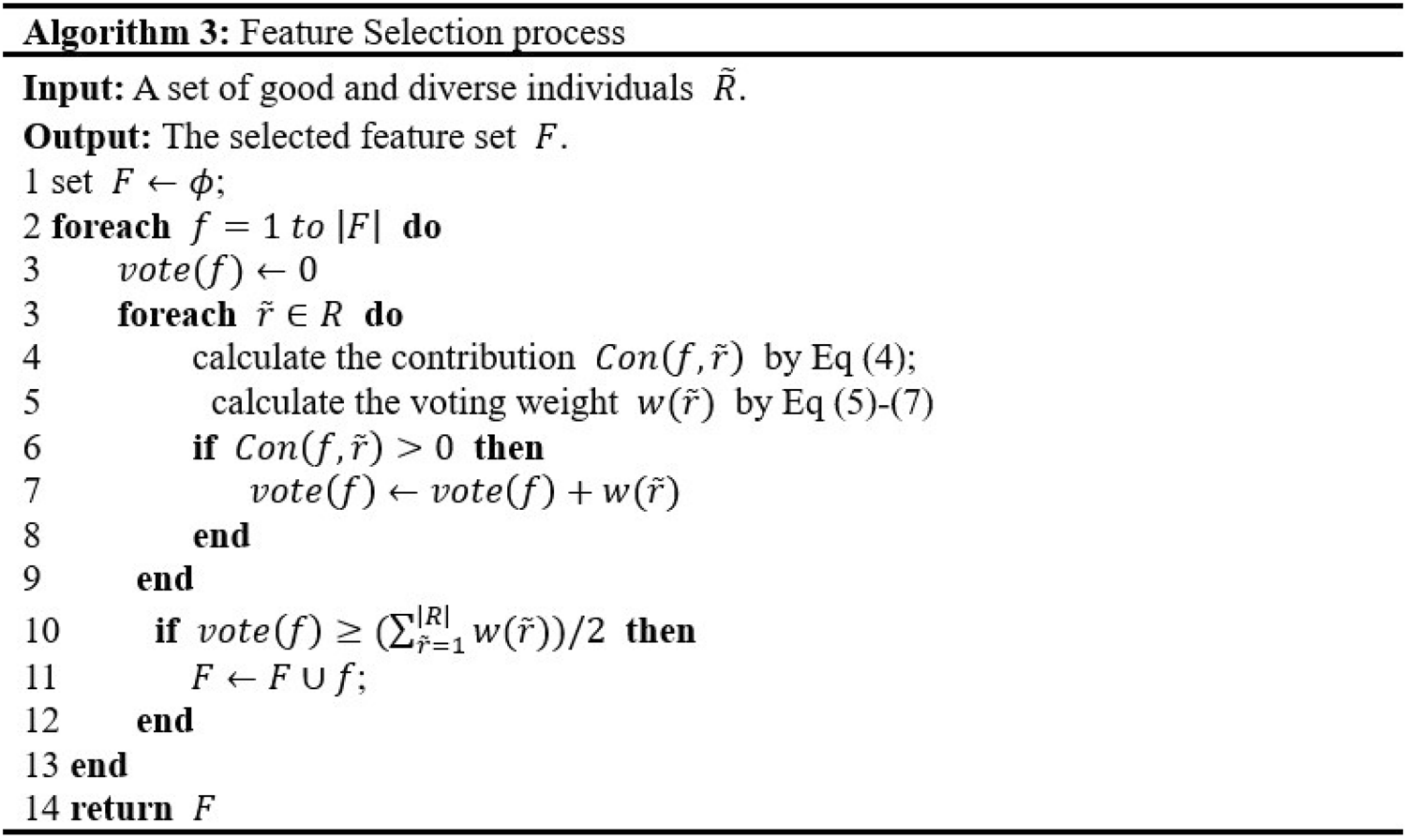


### Individual adaptation strategy

During the third stage, the GP algorithm is employed alongside the selected features to facilitate the development of superior and understandable rules using promising individuals. Nevertheless, there exist unchosen features within the final population. Previous studies have proposed two representative individual adaptation strategies to tackle this issue^45^. A prevalent strategy is to assign a constant value of one to the unselected feature in the rule. One approach involves the replacement of individuals in the final population during stage one based on selected features, ensuring that only phenotypically similar individuals are retained. The findings derived from their study indicate that both approaches are proficient in acquiring information from the final population. The findings indicate that the first strategy exhibits greater potential for inheritance compared to the second. Thus, to decrease the computational expenses associated with producing behaviorally similar individuals, the initial approach involves discarding unchosen features while preserving the individual's structure to the greatest extent feasible. The third phase of the process utilizes the conventional GP algorithm, with the exception of the initialization and mutation techniques. In the process of initialization, the original population is partitioned into two distinct parts. The first part encompasses prospective candidates produced by the innovative GP algorithm, whereas the latter part encompasses random candidates generated by the chosen attributes. The application of the standard subtree mutation involves the generation of randomized trees that exclusively utilize the chosen features.

## Experimental design

### Discrete event simulation model

Previously, a number of GP-HH techniques for DJSS have been evaluated using discrete event-based simulations^43,48^. The simulation parameters employed in this experimental setup is given as follows:

- The model simulates a job shop comprising of 10 machines.
- The arrival of jobs at the shop is a dynamic process that conforms to the Poisson distribution.
- A period of 500 jobs is necessary for the system to reach a stable state before the data from the subsequent 2000 jobs can be utilized.
- Each job comprises a sequence of 2 to 10 distinct operations.
- The average processing time of each operation is sampled from a uniform distribution with a lower bound of 25 and an upper bound of 100.
- Jobs are given weights 1, 2, or 4 with probability 0.2, 0.6, and 0.2.
- The due dates for jobs are determined using the Total Work Content approach, which incorporates different levels of tightness factors.

It is important to create a broad variety of scenarios representing various issue cases in order to evaluate the effectiveness of the rules that have been developed. According to earlier research, the tightness factor and machine usage are the crucial variables utilized to establish load circumstances, which have a major impact on rule performance^29^. Both low and heavy load cases are considered in this paper to evaluate the effectiveness of the developed dispatching rules. To do this, two or three values are set for α and μ in the job-shop simulation, ranging from 2 to 7 and 80% to 99%, respectively. During the training phase, each scenario in a training set is processed once by the simulator. In order to obtain accurate results from the simulation, 20 simulated replications are run to test the created rules. Table 2 details the parameters of the simulation scenarios represented by the tuple $\langle \overline{p }, \alpha , \mu \rangle$.

**Table 2:** Scenarios used in simulations for training and testing.

Parameter	Description	Training	Test
$\overline{p }$	Mean processing time	25, 50	25, 50, 100
$\alpha$	Due dates tightness factor	3, 5, 7	2, 4, 6
$\mu$	Shop utilization level	85, 90, 95	80, 90, 99,
Scenarios $\times$ replications		18 $\times$ 1	27 $\times 20$

Moreover, when evaluating the overall performance of each dispatching rules $r$, the fitness function is calculated by Eq. (19), where $f\left(r,s\right)$ is the value of scheduling objective, which is calculated by applying the rule $r$ to a training instance $s\in S$, ${f}_{ref}\left(s\right)$ denotes the target value, obtained by the reference rule in the same training instance. The best rule for a training instance $s$ through all iterations is considered as refence rule in this article.

19 $$fitness\left(r\right)=\frac{1}{\left|S\right|}{\sum }_{s=1}^{\left|S\right|}\frac{f\left(r,s\right)}{{f}_{ref}\left(s\right)}.$$

### Algorithm parameters

The Table 3 presents a comprehensive list of the terminal and function sets. The set of terminals utilized in the experiment comprises the typical characteristics employed in the existing research concerning GP-HH methodologies^44,45,49^. These features include a variety of aspects, including those related to jobs, machines, and workshop. The function set comprises of the four conventional mathematical operators $+, -, \times$, $/$. The operator denoted by “/” is known to perform protected division, wherein the result obtained is one in the event of the denominator being zero. Also, the “max” and “min” functions are used.

**Table 3:** The GP terminal and function sets.

Node name	Description
NOW	The current time
PT	Processing time of the operation
NPT	Processing time of the next operation
OWT	The waiting time of the operation
NOIQ	Number of operations in the current queue
NOINQ	Number of operations in the next queue
WIQ	Work in the current queue
WINQ	Work in the next queue
MRT	Ready time of the machine
ORT	Ready time of the operation
NOR	Number of operations remaining
WKR	Work remaining (including the current operation)
DD	Due date of the job
W	Weight of the job
SL	Slack time of the job
FDD	Flow due date of the operation
Function set	+ , –, × , /, max, min

Table 4 presents additional parameter settings of the algorithm.

**Table 4:** Parameter settings.

Parameter	Value
Initialization	Ramped-half-and-half
Population size	450
Maximal depth	8
Crossover/mutation rate	90%/20%
Selection	Tournament selection (size = 5)
Number of generations in stage 1 and stage 3	50/50
Terminal/non-terminal selection rate	10%/90%

### Comparison design

To substantiate the achievements of the proposed methodology (NGP-FS), three distinct algorithms have been considered for comparative analysis. The present study employs the standard genetic programming algorithm (SGP as the baseline approach, without incorporating feature selection. To examine the effect of the online feature selection mechanism on the regular GP, the SGP with feature selection technique, also known as SGP-FS, is compared. The novel genetic programming (NGP) is also compared to see whether dynamic diversity management in GP without feature selection improves the algorithm's capacity to provide compact and superior dispatching rules. According to the quality of the solutions and the rules' interpretability, all techniques are assessed and compared. Furthermore, an evaluation of the rule performance is conducted in comparison to the benchmark rules presented in Table 5.

**Table 5:** Benchmark dispatching rules.

Benchmark rules	Descriptions
SPT	Shortest processing time
EDD	Earliest due date
FDD	Earliest flow due date
LPT	Longest processing time
FIFO	First in first out
LILO	Last in last out
CR	Critical ratio
RR	Raghu and Rajendran
MDD	Modified due date
SL	Slack
WATC	Weighted apparent tardiness cost
COVERT	Cost over time
PW	Process waiting time
NPT	Next processing time
WINQ	Work in next queue
PT + WINQ	Processing time + WINQ
2PT + WINQ + NPT	Double processing time + WINQ + NPT
PT + WINQ + SL	Processing time + WINQ + SL
SPT + PW + FDD	Processing time + PW + FDD
2PT + WINQ + NPT + WSL	2Processing time + WINQ + NPT + waiting slack

## Results and discussion

As previously stated, the efficacy of the NGP-FS method is evaluated by comparing it to the SGP, SGP-FS, and NGP approaches. The four GP-based approaches are compared using the three major performance indicators of test performance, mean rule length (number of nodes), and computing time. Larger values of the percentage change objective indicate superior performance, whereas lower value of mean rule length and computing time indicate better performance. The Wilcoxon rank sum test is used for statistical significance testing, with a significance threshold of 0.05. The algorithm was coded in Python 3.8, and the tests were conducted on a system with Intel(R) Xeon (R) CPUs at 3.40 GHz and 128 GB of RAM.

### Training performance

**Figure 3:**
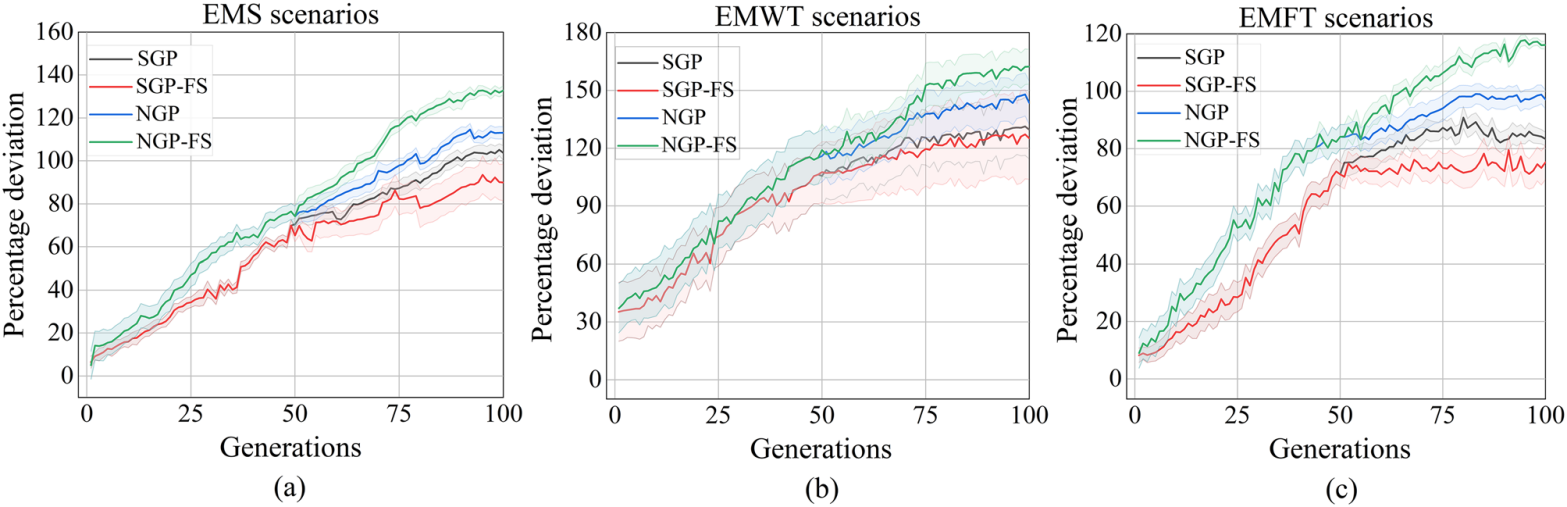
The percentage deviation of the GP algorithms for the EMS, EMWT, and EMFT scenarios in the training stage.

The statistical comparison of the proposed approach NGP-FS with the three algorithms in terms of the three kinds of multiple-objectives is shown in Table 6. The symbols " + ", "–" and " = " within the results indicate that the corresponding outcome is noticeably better than, significantly worse than, or about equal to its counterparts, respectively. The performance of the evolved rule $r$ on a given test is determined by calculating the percentage deviation from the reference rule. This expressed as $100\cdot \left(1-fitness(r)\right)$. Figure 3a–c display the percentage deviation for the EMS, EMWT, and EMFT scenarios.

**Table 6:** Mean and standard deviation of the performance measures (training phase).

Measures	Obj	SGP	SGP-FS	NGP	NGP-FS
Percentage deviation	EMS	105.52 ± 3.73	95.95 ± 10.89	115.66 ± 2.77	131.32 ± 2.21(+ , + , +)
	EMWT	125.82 ± 14.36	122.43 ± 20.41	139.71 ± 11.9	156.58 ± 10.1(+ , + , +)
	EMFT	98.16 ± 5.27	86.27 ± 7.6	111.37 ± 5.97	119.73 ± 4.48(+ , + , +)
Mean rule size	EMS	24.02 ± 2.35	19.63 ± 2.28	18.56 ± 2.87	16.29 ± 3.43(+ , + , +)
	EMWT	26.34 ± 3.28	24.38 ± 3.11	21.09 ± 2.98	17.68 ± 2.56(+ , + , +)
	EMFT	25.04 ± 3.89	20.26 ± 3.71	19.13 ± 1.59	13.43 ± 1.37(+ , + , +)
Computational time	EMS	141.39 ± 3.25	135.52 ± 2.86	155.27 ± 6.58	142.54 ± 3.51(+ , = , +)
	EMWT	199.27 ± 3.63	190.25 ± 4.56	220.24 ± 4.16	205.47 ± 4.14(+ , = , +)
	EMFT	123.42 ± 4.53	120.47 ± 4.25	130.24 ± 2.58	125.48 ± 2.36(+ , = , +)

With regards to the evaluation of rule performance, it has been observed that the NGP-FS algorithm exhibits superior performance compared to all other algorithms for the three objectives that were analyzed. It is noteworthy that the SGP-FS algorithm exhibits poor performance across all scenarios. This result appears to be paradoxical as feature selection is commonly acknowledged as a viable approach to minimize irrelevant characteristics in GP algorithm, thereby improving the overall quality of the solution. The above observation suggests that the accuracy of feature selection may be influenced by the quality of the final population obtained in the first stage, despite the utilization of identical mutation processes and adaptation strategies for both SGP-FS and NGP-FS in the third stage.

**Figure 4:**
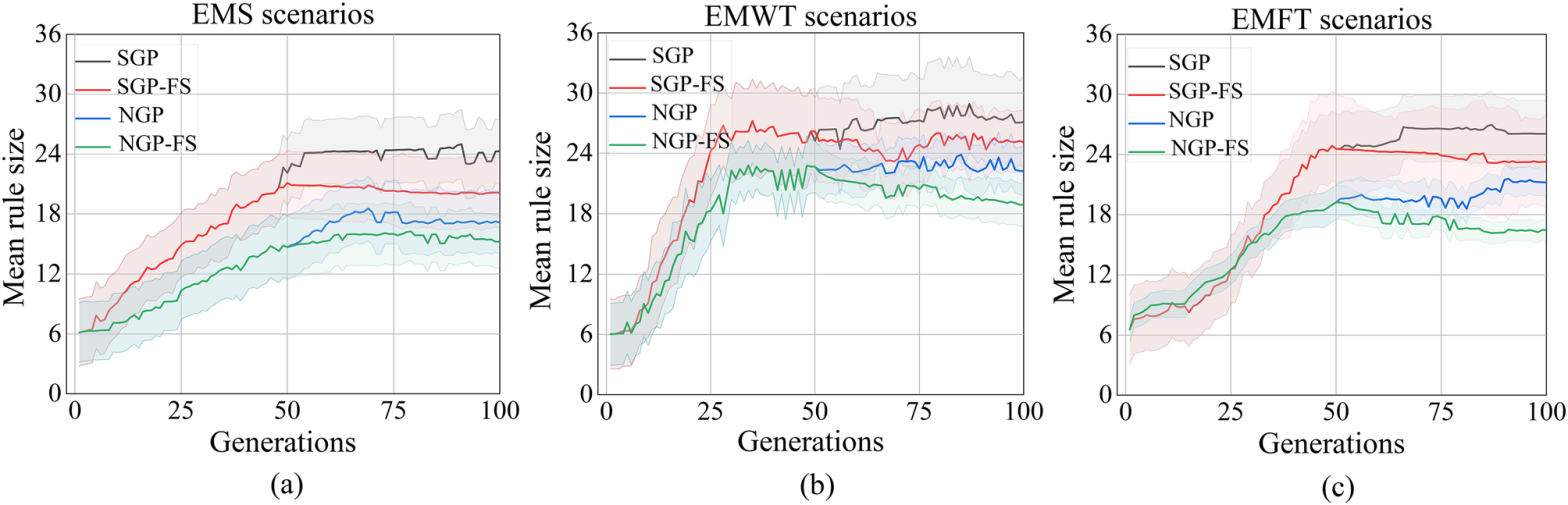
The average rule size of the GP algorithms for the EMS, EMWT, and EMFT scenarios in the training stage.

The development of concise and readily understandable dispatching rules is also a crucial aspect of energy-aware scheduling assignments. The utilization of simple dispatching rules confers benefits in terms of decreased computational costs and heightened generalizability. The changes in the mean rule size across generations are depicted in Fig. 4a–c under the EMS, EMWT, and EMFT, respectively. The results indicate that the SGP algorithm exhibits a tendency to generate significantly larger rules across all evaluated objectives in comparison to other algorithms. The findings are consistent with prior studies that have demonstrated that rules generated by the standard GP algorithm tend to be more extensive. Despite the utilization of the feature selection mechanism for eliminating redundant features in SGP-FS, the average rule size remains greater than that of NGP and NGP-FS across the three objectives. The NGP algorithm exhibits the second-lowest average rule size, indicating that the GP’s rule size is positively influenced by the dynamic management of diversity. Following the feature selection process, specifically after 50 generations, it has been observed that the evolved rules of NGP-FS exhibit greater compactness in comparison to those generated by the NGP. The NGP-FS algorithm has been proposed as a means to achieve small feature subsets while concurrently producing concise rules.

**Figure 5:**
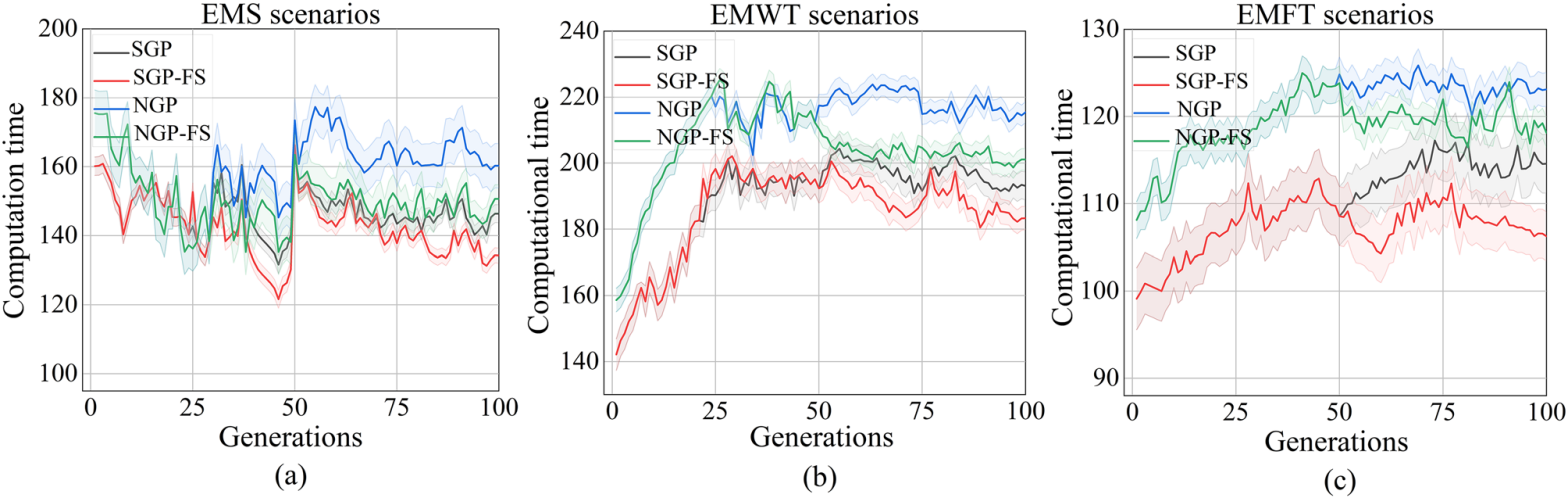
The computational time of the GP algorithms for the EMS, EMWT, and EMFT scenarios in the training stage.

The results depicted in Fig. 5a–c demonstrate that the NGP algorithm requires a greater computational budget compared to the other algorithm across all three scenarios. The primary factor is that the replacement operator demands a greater degree of individual evaluation. Despite the lack of initial advantage in the first 50 generations, the NGP-FS algorithm ultimately demonstrated comparable computational efficiency to both the SGP and SGP-FS algorithms after the feature selection stage. The suggestion put forth is that utilizing a restricted terminal set that includes selected features may result in more efficient rules as opposed to utilizing a vast feature set. Additionally, it should be noted that there is very little variation in the computational times of the SGP and SGP-FS, indicating that a chosen feature without accuracy cannot shorten the algorithm’s time for computation.

In conclusion, it can be inferred that the NGP-FS algorithm has the ability to generate efficient and concise dispatching rules within a reasonable computational timeframe, while also taking into account various scenarios that prioritize energy savings.

### Test performance

This section presents the outcomes of test scenarios for the EMS, EMWT, and EMFT to demonstrate the efficacy of the proposed approach. Table 7a–c present the mean and standard deviation values of the optimal dispatching rules generated by the four algorithms across 30 iterations for three distinct objectives. Tests also show that the objective value of the best benchmarking rules for each scenario. Under the EMS scenarios, it has been observed that the NGP-FS algorithm exhibits superior performance as compared to the BR, SGP, and SGP-FS algorithms across all simulated instances. Especially when the instances increase in complexity, the disparity between them becomes more apparent, e.g. The benchmark rule 2PT + WINQ + NPT yielded an objective value that was 200% greater than the objective values obtained by the rules generated through NGP-FS in scenarios with parameters < 100, 2, 99% > and < 50, 2, 99% > . In 24 instances, NGP-FS yielded superior EMS outcomes in comparison to the NGP algorithm, while in 4 instances, no significant difference was observed. The rules generated through the utilization of the SGP-FS algorithm exhibit inferior performance in comparison to the rules formulated by other algorithms and benchmark rules.

**Table 7:** Mean and standard deviation of the (a) EMS, (b) EMWT and (c) EMFT objective value of the considered algorithms in the testing phase.

Scenarios	RBR	SGP	SGP-FS	NGP	NGP-FS
(a) Mean and standard deviation of the EMS objective value of the considered algorithms in the testing phase					
(20, 2, 80%)	857.25 ± 12.79 (PT + WINQ)	854.14 ± 8.21	858.75 ± 5.66	846.37 ± 8.42	842.81 ± 6.17 (+ , + , + , +)
(20, 2, 90%)	956.12 ± 20.25 (2PT + NPT + WINQ)	929.57 ± 10.27	939.65 ± 7.38	925.31 ± 18.25	924.54 ± 19.77 (+ , + , + , +)
(20, 2, 99%)	813.15 ± 20.13 (PT + WINQ)	825.34 ± 10.56	837.57 ± 16.47	828.34 ± 13.34	734.88 ± 4.58 (+ , + , + , +)
(20, 4, 80%)	892.34 ± 16.57 (PT + WINQ)	893.12 ± 11.58	897.34 ± 14.16	891.27 ± 13.78	887.24 ± 12.24 (+ , + , + , +)
(20, 4, 90%)	920.57 ± 25.37 (PT + WINQ)	921.63 ± 17.41	925.58 ± 18.34	915.67 ± 27.81	916.52 ± 28.61(+ , + , + , =)
(20, 4, 99%)	1129.34 ± 15.22 (COVERT)	1122.34 ± 3.3	1138.85 ± 25.15	1113.78 ± 18.67	1114.45 ± 15.28 (+ , + , + , =)
(20, 6, 80%)	935.37 ± 21.09 (COVERT)	927.27 ± 11.57	933.58 ± 8.18	925.38 ± 17.35	921.21 ± 15.29 (+ , + , + , +)
(20, 6, 90%)	898.25 ± 14.37 (PT + WINQ)	897.37 ± 11.24	899.62 ± 10.48	894.87 ± 15.16	892.54 ± 12.38 (+ , + , + , =)
(20, 6, 99%)	917.25 ± 25.02 (SL/RO)	918.66 ± 15.27	919.27 ± 18.34	913.28 ± 12.28	915.37 ± 10.48 (+ , + , + , +)
(50, 2, 80%)	884.28 ± 18.62 (PT + WINQ)	883.24 ± 4.69	885.19 ± 5.16	878.32 ± 5.15	875.27 ± 4.29 (+ , + , + , +)
(50, 2, 90%)	975.51 ± 15.92 (PT + WINQ)	969.17 ± 8.56	972.29 ± 9.71	969.19 ± 4.31	965.18 ± 5.57 (+ , + , + , +)
(50, 2, 99%)	1236.24 ± 56.21 (2PT + NPT + WINQ)	1223.78 ± 13.34	1245.56 ± 45.75	1175.73 ± 11.34	1115.48 ± 12.15 (+ , + , + , +)
(50, 4, 80%)	847.25 ± 13.15 (2PT + NPT + WINQ)	847.37 ± 5.85	851.34 ± 10.62	837.14 ± 6.75	834.28 ± 5.23 (+ , + , + , +)
(50, 4, 90%)	803.5 ± 38.27 (COVERT)	799.45 ± 20.26	810.26 ± 25.15	787.12 ± 20.10	785.14 ± 15.34 (+ , + , + , =)
(50, 4, 99%)	877.97 ± 20.25 (RR)	875.15 ± 10.63	885.84 ± 12.16	880.63 ± 7.27	867.23 ± 10.34 (+ , + , + , +)
(50, 6, 80%)	848.89 ± 30.45 (PT + WINQ)	845.52 ± 14.06	856.29 ± 13.81	842.87 ± 11.93	837.65 ± 14.34 (+ , + , + , +)
(50, 6, 90%)	867.34 ± 19.78 (SL/RO)	869.29 ± 14.43	872.37 ± 11.67	860.25 ± 12.29	854.34 ± 13.26 (+ , + , + , +)
(50, 6, 99%)	875.35 ± 13.38 (COVERT)	878.67 ± 7.88	882.34 ± 8.13	869.65 ± 6.08	864.21 ± 5.12 (+ , + , + , +)
(100, 2, 80%)	961.67 ± 7.14 (2PT + NPT + WINQ)	958.97 ± 5.76	963.79 ± 5.28	941.18 ± 4.47	865.28 ± 3.68 (+ , + , + , +)
(100, 2, 90%)	941.25 ± 25.94 (PT + WINQ)	938.15 ± 18.13	945.17 ± 17.99	926.48 ± 17.79	917.26 ± 16.52 (+ , + , + , +)
(100, 2, 99%)	947.57 ± 22.46 (2PT + NPT + WINQ)	942.68 ± 13.57	952.49 ± 26.14	905.37 ± 12.21	853.23 ± 14.13 (+ , + , + , +)
(100, 4, 80%)	935.54 ± 25.65 (PT + WINQ)	934.32 ± 3.48	940.14 ± 8.27	927.37 ± 6.28	871.57 ± 4.67 (+ , + , + , +)
(100, 4, 90%)	842.83 ± 32.71 (COVERT)	840.18 ± 25.06	851.29 ± 21.28	829.16 ± 20.42	825.84 ± 20.28 (+ , + , + , +)
(100, 4, 99%)	1301.55 ± 10.68 (PT + WINQ)	1283.67 ± 15.47	1302.47 ± 22.09	1162.28 ± 15.13	1056.42 ± 14.67 (+ , + , + , +)
(100, 6, 80%)	827.26 ± 43.24 (COVERT)	822.71 ± 25.16	832.71 ± 36.30	811.06 ± 30.95	898.08 ± 32.16 (+ , + , + , +)
(100, 6, 90%)	898.05 ± 75.36 (COVERT)	895.78 ± 14.26	815.48 ± 24.21	887.26 ± 18.81	881.27 ± 18.28 (+ , + , + , +)
(100, 6, 99%)	842.34 ± 25.38 (SL/RO)	837.85 ± 4.87	849.92 ± 13.51	834.91 ± 8.29	832.35 ± 6.45 (+ , + , + , +)
(b) Mean and standard deviation of the EMWT objective value of the considered algorithms in the testing phase					
(20, 2, 80%)	1783.27 ± 85.48 (WATC)	1726.94 ± 87.61	1794.65 ± 121.26	1789 ± 99.34	1368.67 ± 71.27 (+ , + , + , +)
(20, 2, 90%)	2389.42 ± 68.15 (PT + WINQ)	2374.36 ± 68.27	2383.19 ± 68.67	2364.22 ± 67.27	2329.45 ± 48.38 (+ , + , + , +)
(20, 2, 99%)	2924.32 ± 78.31 (WATC)	2914.53 ± 85.25	2945.96 ± 98.67	2847.29 ± 105.28	2515.84 ± 23.56 (+ , + , + , +)
(20, 4, 80%)	2687.66 ± 34.98 (WATC)	2699.39 ± 32.43	2713.47 ± 44.21	2616.48 ± 48.16	2459.24 ± 29.13 (+ , + , + , +)
(20, 4, 90%)	3796.54 ± 19.58 (2PT + NPT + WINQ)	3834.67 ± 22.15	3867.49 ± 24.14	3791.94 ± 20.36	3579.29 ± 25.41 (+ , + , + , +)
(20, 4, 99%)	3948.76 ± 25.48 (PT + WINQ)	3951.48 ± 20.95	3995.37 ± 28.31	3927.64 ± 33.78	3475.24 ± 20.28 (+ , + , + , +)
(20, 6, 80%)	3589.76 ± 21.79 (COVERT)	3607.38 ± 24.13	3635.07 ± 32.95	3665.67 ± 28.37	3637.85 ± 27.12 (+ , + , + , +)
(20, 6, 90%)	3837.95 ± 40.15 (COVERT)	3825.11 ± 50.48	3837.59 ± 64.37	3812.48 ± 75.05	3858.24 ± 64.27 (+ , + , + , +)
(20, 6, 99%)	2795.61 ± 16.47(RR)	2784.29 ± 11.23	2894.82 ± 17.89	2737.33 ± 9.34	2331.11 ± 8.97 (+ , + , + , +)
(50, 2, 80%)	2484.58 ± 16.87 (PT + WINQ)	2413.41 ± 15.26	2485.29 ± 2321	2378.52 ± 14.35	1957.39 ± 15.21 (+ , + , + , +)
(50, 2, 90%)	1768.52 ± 24.19 (PT + WINQ)	1769.37 ± 25.48	1872.94 ± 47.58	1699.31 ± 23.57	1575.28 ± 12.96 (+ , + , + , +)
(50, 2, 99%)	2395.77 ± 18.59 (COVERT)	2395.56 ± 39.71	2428.27 ± 41.82	2385.52 ± 27.65	1835.56 ± 19.24 (+ , + , + , +)
(50, 4, 80%)	4558.98 ± 21.44 (2PT + NPT + WINQ)	4607.54 ± 21.64	4692.23 ± 28.26	4548.12 ± 24.19	4435.61 ± 24.24 (+ , + , + , +)
(50, 4, 90%)	2514.67 ± 32.78 (COVERT)	2519.53 ± 37.79	2520.28 ± 35.27	2487.55 ± 31.72	2399.47 ± 25.67 (+ , + , + , +)
(50, 4, 99%)	3256.67 ± 19.74 (SL/RO)	3255.25 ± 19.63	3267.64 ± 21.26	3150.47 ± 18.25	3138.33 ± 19.31 (+ , + , + , +)
(50, 6, 80%)	2683.89 ± 12.21 (PT + WINQ)	2645.12 ± 15.17	2658.29 ± 18.35	2799.28 ± 13.72	2037.45 ± 12.13 (+ , + , + , +)
(50, 6, 90%)	3667.31 ± 30.38 (SL/RO)	3660.37 ± 31.83	3665.37 ± 28.67	3560.25 ± 42.69	3454.15 ± 28.35 (+ , + , + , +)
(50, 6, 99%)	1972.59 ± 14.72 (COVERT)	1989.37 ± 15.18	982.16 ± 11.23	869.45 ± 6.27	689.25 ± 8.74 (+ , + , + , +)
(100, 2, 80%)	3651.37 ± 15.18 (2PT + NPT + WINQ)	3648.26 ± 15.24	3686.81 ± 15.27	3531.48 ± 34.61	3225.76 ± 15.28 (+ , + , + , +)
(100, 2, 90%)	3526.65 ± 26.18 (PT + WINQ)	3518.15 ± 27.13	3545.45 ± 24.99	3326.21 ± 28.81	3117.46 ± 26.42 (+ , + , + , +)
(100, 2, 99%)	2466.67 ± 15.86 (2PT + NPT + WINQ)	2452.68 ± 15.29	2487.59 ± 17.21	2385.71 ± 16.21	1983.65 ± 10.13 (+ , + , + , +)
(100, 4, 80%)	2530.64 ± 24.61 (PT + WINQ)	2529.32 ± 20.35	2635.74 ± 22.59	2482.77 ± 27.14	2415.57 ± 18.27 (+ , + , + , +)
(100, 4, 90%)	2442.83 ± 53.92 (COVERT)	2483.58 ± 57.06	2541.29 ± 58.47	2429.56 ± 42.47	2285.95 ± 41.18 (+ , + , + , +)
(100, 4, 99%)	2539.55 ± 33.37 (PT + WINQ)	2533.17 ± 35.28	2618.37 ± 34.29	2429.78 ± 37.53	2176.42 ± 35.87 (+ , + , + , +)
(100, 6, 80%)	1927.16 ± 16.05 (COVERT)	1928.81 ± 18.26	1999.87 ± 18.27	1911.26 ± 12.96	1618.48 ± 14.36 (+ , + , + , +)
(100, 6, 90%)	2798.25 ± 18.17 (COVERT)	2795.78 ± 15.19	2815.48 ± 20.38	2687.26 ± 16.71	2481.47 ± 10.54 (+ , + , + , +)
(100, 6, 99%)	2922.54 ± 37.56 (COVERT)	2917.85 ± 34.73	3149.92 ± 33.74	2834.91 ± 28.29	2232.55 ± 17.34 (+ , + , + , +)
(c) Mean and standard deviation of the EMFT objective value of the considered algorithms in the testing phase					
(20, 2, 80%)	461.21 ± 9.45 (PT + WINQ)	455.74 ± 3.07	459.27 ± 3.14	451.35 ± 2.37	435.57 ± 2.76 (+ , + , + , +)
(20, 2, 90%)	442.89 ± 11.25 (2PT + NPT + WINQ)	438.68 ± 4.57	441.56 ± 5.34	433.63 ± 4.21	422.28 ± 3.58 (+ , + , + , +)
(20, 2, 99%)	455.33 ± 12.42 (PT + WINQ)	355.68 ± 5.64	351.61 ± 5.39	315.27 ± 4.59	318.25 ± 3.31 (+ , + , + , +)
(20, 4, 80%)	394.23 ± 7.19 (PT + WINQ	388.57 ± 5.14	392.25 ± 5.29	386.63 ± 3.13	381.23 ± 3.17 (+ , + , + , +)
(20, 4, 90%)	438.62 ± 10.86 (PT + WINQ)	431.18 ± 5.42	434.69 ± 8.92	428.37 ± 7.65	422.42 ± 7.64 (+ , + , + , +)
(20, 4, 99%)	498.67 ± 15.38 (PT + WINQ)	489.28 ± 7.13	494.23 ± 6.13	487.48 ± 5.17	465.15 ± 3.27 (+ , + , + , +)
(20, 6, 80%)	328.52 ± 13.97 (PT + WINQ)	329.15 ± 4.49	339.60 ± 5.27	333.75 ± 3.15	328.75 ± 4.08 (+ , + , + , +)
(20, 6, 90%)	405.79 ± 15.28 (PT + WINQ)	396.33 ± 16.01	401.75 ± 6.27	494.67 ± 6.15	485.84 ± 3.47 (+ , + , + , +)
(20, 6, 99%)	634.84 ± 28.76 (PT + WINQ)	630.52 ± 6.35	633.34 ± 8.48	716.57 ± 4.17	556.21 ± 3.12 (+ , + , + , +)
(50, 2, 80%)	451.85 ± 11.34 (PT + WINQ)	441.25 ± 6.93	447.69 ± 6.09	435.12 ± 6.25	425.26 ± 6.26 (+ , + , + , +)
(50, 2, 90%)	589.62 ± 15.05 (PT + WINQ)	588.23 ± 5.57	585.49 ± 5.25	586.19 ± 2.74	575.38 ± 2.11 (+ , + , + , +)
(50, 2, 99%)	695.27 ± 34.23 (2PT + NPT + WINQ)	687.36 ± 6.24	691.21 ± 5.32	685.12 ± 3.71	635.18 ± 3.27 (+ , + , + , +)
(50, 4, 80%)	517.98 ± 45.24 (2PT + NPT + WINQ)	498.26 ± 4.95	515.23 ± 4.17	485.15 ± 3.94	480.69 ± 2.76 (+ , + , + , +)
(50, 4, 90%)	531.16 ± 48.28 (PT + WINQ)	525.15 ± 5.79	528.15 ± 6.08	521.67 ± 4.88	512.66 ± 2.89 (+ , + , + , +)
(50, 4, 99%)	727.57 ± 59.25 (PT + WINQ)	724.27 ± 9.73	725.24 ± 10.16	691.23 ± 7.13	628.58 ± 4.15 (+ , + , + , +)
(50, 6, 80%)	458.19 ± 12.79 (PT + WINQ)	448.12 ± 8.15	456.29 ± 8.91	445.81 ± 8.12	437.25 ± 6.21 (+ , + , + , +)
(50, 6, 90%)	597.11 ± 31.67 (PT + WINQ)	592.29 ± 14.43	596.17 ± 9.23	585.36 ± 7.19	574.37 ± 6.28 (+ , + , + , +)
(50, 6, 99%)	795.25 ± 67.36 (2PT + NPT + WINQ)	789.37 ± 6.98	793.26 ± 7.27	786.35 ± 5.98	708.48 ± 5.12 (+ , + , + , +)
(100, 2, 80%)	581.27 ± 45.48 (2PT + NPT + WINQ)	574.21 ± 6.27	583.29 ± 6.18	586.18 ± 5.62	575.86 ± 3.24 (+ , + , + , +)
(100, 2, 90%)	741.55 ± 56.34 (PT + WINQ)	732.25 ± 7.27	738.47 ± 7.25	726.58 ± 6.71	718.46 ± 4.23 (+ , + , + , +)
(100, 2, 99%)	932.17 ± 84.52 (2PT + NPT + WINQ)	912.38 ± 3.59	926.19 ± 4.23	898.27 ± 4.62	813.25 ± 2.73 (+ , + , + , +)
(100, 4, 80%)	665.27 ± 43.35 (PT + WINQ)	651.12 ± 6.34	665.24 ± 6.15	640.27 ± 4.21	645.28 ± 4.20 (+ , + , + , +)
(100, 4, 90%)	653.83 ± 62.71 (2PT + NPT + WINQ)	646.18 ± 7.08	651.29 ± 8.23	639.26 ± 7.12	627.34 ± 6.18 (+ , + , + , +)
(100, 4, 99%)	915.34 ± 82.56 (PT + WINQ)	908.27 ± 8.26	912.47 ± 8.09	904.28 ± 7.58	856.12 ± 7.12 (+ , + , + , +)
(100, 6, 80%)	637.45 ± 76.19 (PT + WINQ)	628.31 ± 8.09	632.78 ± 8.31	621.76 ± 7.25	598.48 ± 5.52 (+ , + , + , +)
(100, 6, 90%)	768.34 ± 76.16 (PT + WINQ)	761.78 ± 14.27	765.25 ± 24.11	757.26 ± 8.23	725.17 ± 8.56 (+ , + , + , +)
(100, 6, 99%)	956.54 ± 98.38 (PT + WINQ)	938.75 ± 7.48	952.92 ± 8.35	928.11 ± 7.34	858.16 ± 5.02 (+ , + , + , +)

In relation to the EMWT objective, the NGP-FS algorithm demonstrated superior performance in comparison to other methods across all 27 instances. It is notable to state that the difference in the efficacy of the NGP-FS algorithm is more pronounced in comparison to the other algorithms. In situations characterized by higher shop utilization rates and strict deadlines, the NGP-FS algorithm yields superior outcomes compared to the NGP and SGP. This indicates that the NGP-FS can generate rules that are competitive while adhering to the time restrictions. As anticipated, the rules created by SGP-FS exhibit inferior solution quality compared to the outcomes achieved by the remaining algorithms across all scenarios.

For the EMFT scenarios, according to Table 7c, the NGP-FS method continues to exhibit the highest objective values among the methods under consideration. However, the gap in rule performance is not deemed significant when compared to scenarios featuring MT and MWT objectives. Furthermore, the differences become greater in situations where there is a high level of shop utilization and a significant tightness factor in comparison to the other scenarios.

The experiment results reveal the subsequent findings: (1) The results indicate that the GP based methods outperform the manually designed dispatching rules in terms of robustness in the energy aware scheduling, as evidenced by the lower standard deviations achieved. Specifically, artificial dispatching rules exhibit inconsistency in their outcomes when applied across diverse working conditions. (2) When dealing with various job shop settings, the suggested NGP-FS algorithm can produce high-quality rules with amazing small sizes in a reasonable computational time when compared to SGP, SGP-FS, NGP, and benchmark rules. (3) The objective values increase with the job shop scenarios become more complex under three objectives, which indicates that the complexity of workshop shows major impact on the energy aware scheduling while the processing time, standby power and processing power owns minor influence.

## Feature analysis

### Feature analyses

**Figure 6:**
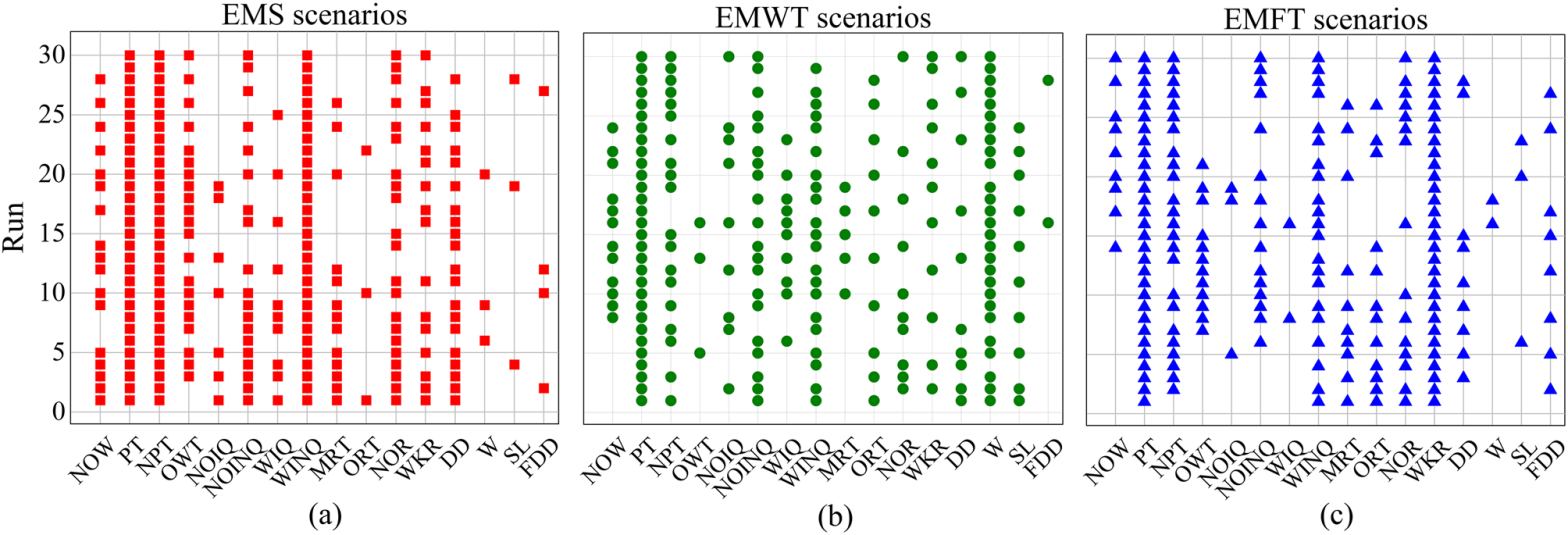
The matrix plot of the feature selection results of the NGP-FS Algorithm for the EMS, EMWT, and EMFT scenarios.

The results of feature selection for the EMS, EMWT, and EMFT scenarios using the NGP-FS algorithm are presented in Fig. 6a–c, respectively. The data shown in the figures are based on 30 independent runs. Each row of the matrix represents a run, while each column represents a feature. A point is drawn at the intersection of the selected feature $f$ and the $i$ th run. For the EMS scenario, the feature PT, NPT, and WKR are selected in all 30 runs. This indicates that the processing time of operations in the EMS scenario has an important impact on the computation of job priorities. It is evident that minimizing idle times on machines with high unload power would be advantageous when allocating tasks to machines. This trade-off could potentially result in a longer makespan and an uneven distribution of idle periods. In the meantime, the reduction of makepan has the potential to result in schedules characterized by decreased idle durations. In order to improve machine utilization, the idle times generated by the designed rules may be allocated equally across each machine. So, the machine prefers to select jobs with short processing time, less remaining processes, and low processing power. Moreover, the features NOINQ, NOR, and OWT are selected in more than half of the running times of the algorithm, indicating that they significantly contribute to the generation of optimal dispatching rules at least half of the time. It can also be seen that the feature FDD, W, and SL are selected only a few times, which indicates that these features are irrelevant or redundant in this scenario and may not contribute to the generation of best dispatching rules.

Figure 6b exhibits that PT and W are the most important features to reduce the EMWT scenarios. With the exception of these two features, WIQN, NOINQ, and NPT are likewise chosen in the majority of runs, indicating the importance of the workload information in the next queue to decreasing the EMWT scenarios. The fact that MRT, OWT, and FDD are often not chosen suggests that they have little bearing on the best developed rules for the EMWT. In contrast to the results of previous studies^44^, it has been observed that the attribute DD is not excluded from the irrelevant feature sets, and is chosen in over 40% of instances. This may be due to the scenarios considered energy consumption.

The significance of PT, NPT, WINQ, and WKR in relation to the EMFT scenarios is illustrated in Fig. 6c. The results indicate that PT and WKR were chosen consistently across all 30 runs, suggesting that jobs characterized by shorter processing times and lower remaining workloads are more likely to be prioritized for early processing. The selection of WINQ and NPT in the majority of runs suggests that the workload information in the subsequent queue is a crucial determinant for achieving the objective of the EMFT. The infrequent selection of WIQ, NOIQ, SL, and W features suggests that these characteristics may be redundant or irrelevant in EMFT scenarios and may not significantly contribute to the development of optimal evolved rules.

### Rule analysis

This study employs the numerical reduction technique as described by Nguyen^43^ to simplify the rules and enhance our understanding of their complexity and interpretability. The EMWT scenario is often used as an illustrative example due to its comparatively greater complexity in optimization as compared to other scenarios. The depth, size, and leaf size of the best rules as determined by 30 different runs of the four algorithms in the EMWT scenarios are shown in Table 8 along with their average and standard deviation. According to prior analysis, the algorithm based on NGP exhibits a significantly greater advantage than the algorithm based on SGP with respect to regular structure. Equations (20) and (21) illustrate two distinct rules derived by NGP and NGP-FS for comparison. It should be noted that the size of rule 2 is smaller than that of rule 1. By way of comparison, it can be observed that rule 1 incorporates certain attributes, namely NOR, WIQ, and FDD, which are not deemed to be primary features. This suggests that the contribution of the actual component to the priority function in rule 1 is relatively less significant than that in rule 2. This could be the cause of rule 1’s inferior performance on training tests to rule 2's. The NGP-FS technique, as proposed, effectively identifies crucial building blocks in comparison to the NGP approach by utilizing the vital attribute set. Ultimately, this leads to improved outcomes.

20 $$\begin{aligned} r_{1} & = (WIQ{ + }NPT + FDD{)} \times \frac{NOR}{W} + \max (AT,PT \times NPT) \nonumber\\ &\quad + NOPS + 2DD \times \min (\frac{{SL^{2} }}{NOR + PT}) - MRT{ , } \end{aligned}$$

21 $$r_{2} = \frac{{\max \left( {WINQ + PT,NPT} \right)}}{W} + \max \left( {\frac{PT}{W},\frac{WINQ}{W} + NOR} \right).$$

**Table 8:** Mean and standard deviation of the depth, size and leaves of the best rules obtained by the 30 runs of the four algorithms in the EMWT scenarios.

Algorithm	Depth	Size	Leaves
SGP	7.7 ± 0.37	65.25 ± 20.13	33.28 ± 9.75
SGP-FS	7.6 ± 0.35	54.43 ± 18.67	32.17 ± 9.58
NGP	7.2 ± 0.28	19.34 ± 5.34	10.23 ± 2.18
NGP-FS	6.8 ± 0.34	16.56 ± 4.64	7.23 ± 1.57

## Conclusions and future work

The purpose of this study is to provide an integrative strategy to solve the energy-aware dynamic job shop scheduling issue that combines a novel genetic programming algorithm with feature selection (NGP-FS). This integrated approach used a diversity management technique for the GP algorithm to speed up the search process and enhance rule quality. Moreover, utilizing the feature selection technique, simpler and competitive rules with just significant features were developed. In this study, the NGP-FS approach was evaluated against three other algorithms (SGP, SGP-FS, NGP) in the context of energy consumption scenarios. The comparison was conducted based on three criteria: rule size, quality of designed rules, and computation time. Experimental results demonstrate that the proposed method can generate more interpretable and high-quality rules for EDJSS, as well as accomplish high robustness against complex scenarios. The analysis of rules indicates that the NGP-FS possesses the capability to identify more significant building blocks for enhancing rule performance.

The suggested approach will be used in further research to analyze field datasets obtained from real-world manufacturing systems. The study aims to investigate the utilization of new rules embedded within field datasets as a means of addressing practical job-shop scheduling issues.

## Supplementary Information

Supplementary Information.

## Data Availability

All data generated or analyzed during this study are included in this published article.
