# Supplementary material for: Learning dispatching rules via novel genetic programming with feature selection in energy-aware dynamic job-shop scheduling
Source: Sci Rep. 2023 May 26;13:8558. doi: 10.1038/s41598-023-34951-w (PMC10219999; doi:10.1038/s41598-023-34951-w)
Supplement: Supplementary file 1 — Supplementary Information. [file 41598_2023_34951_MOESM1_ESM.zip › The whole algorithm of the proposed approach.docx]

The pseudo-code of the novel GP algorithm with feature selection is described in Algorithm 4.

| Algorithm 4:The novel GP algorithm with feature selection |
| --- |
| Input: A number of problem instances in training set $\left\{ \boldsymbol{s}_{\boldsymbol{1}}\boldsymbol{,}\boldsymbol{s}_{\boldsymbol{1}}\boldsymbol{,}\boldsymbol{s}_{\boldsymbol{1}}\boldsymbol{,\cdots}\boldsymbol{s}_{\boldsymbol{1}} \right\}\boldsymbol{\in S}$ |
| Output: the best-evolved rule $\boldsymbol{r}_{\boldsymbol{best}}$ |
| 1 $\boldsymbol{P}_{\boldsymbol{0}}$= population-initialization ($\boldsymbol{r}_{\boldsymbol{1}}\boldsymbol{,}\boldsymbol{r}_{\boldsymbol{1}}\boldsymbol{,\cdots,}\boldsymbol{r}_{\boldsymbol{n}}\boldsymbol{)}$ |
| 2 Set $\boldsymbol{r}_{\boldsymbol{best}}\boldsymbol{\leftarrow null}$ and the best fitness value $\boldsymbol{f}\left( \boldsymbol{r}_{\boldsymbol{best}} \right)\boldsymbol{\leftarrow+\infty}$ |
| 3 $\boldsymbol{gen\leftarrow0}$  4 while $\boldsymbol{gen<maxGen}$ do |
| 5 while $\boldsymbol{gen<max}\boldsymbol{Gen}_{\boldsymbol{1}}$ do |
| 6 for all $\boldsymbol{r}_{\boldsymbol{i}}\boldsymbol{\in}\boldsymbol{P}_{\boldsymbol{g}}$ do |
| 7 evaluate $\boldsymbol{f}\left( \boldsymbol{r}_{\boldsymbol{i}} \right)$ by applying to each scenario $\boldsymbol{s}_{\boldsymbol{i}}\boldsymbol{\in S}$ |
| 8 if $\boldsymbol{f}\left( \boldsymbol{r}_{\boldsymbol{i}} \right)\boldsymbol{< f}\left( \boldsymbol{r}_{\boldsymbol{best}} \right)$ then  9 $\boldsymbol{r}_{\boldsymbol{best}}\boldsymbol{\leftarrow}\boldsymbol{r}_{\boldsymbol{i}}$ |
| 10 end if |
| 11 end for |
| 12 if $\boldsymbol{gen<max}\boldsymbol{Gen}_{\boldsymbol{1}}\boldsymbol{-1}$ then |
| 13 $\boldsymbol{P}_{\boldsymbol{s}}$= selection $\boldsymbol{(}\boldsymbol{P}_{\boldsymbol{g}}\boldsymbol{)}$  14 offspring$\boldsymbol{O}^{\boldsymbol{'}}$= crossover $\boldsymbol{P}_{\boldsymbol{s}}$  15 $\boldsymbol{O}$= mutation $\boldsymbol{(}\boldsymbol{O}^{\boldsymbol{'}}\boldsymbol{)}$  16 $\boldsymbol{P}_{\boldsymbol{g}}$= replacement $\boldsymbol{(}\boldsymbol{P}_{\boldsymbol{g}}\boldsymbol{,O)}$  17 $\boldsymbol{C}\boldsymbol{\leftarrow}\boldsymbol{P}_{\boldsymbol{g}}\boldsymbol{\cup}\boldsymbol{O}$  18 $\boldsymbol{P}_{\boldsymbol{new}}\boldsymbol{\leftarrow}\left\{ \boldsymbol{BestIndividual}\left( \boldsymbol{C} \right) \right\}$  19 $\boldsymbol{C}\boldsymbol{\leftarrow}\boldsymbol{C}\boldsymbol{\backslash}\boldsymbol{P}_{\boldsymbol{new}}$  20 $\boldsymbol{D}\mathbf{=}\boldsymbol{minimum} \boldsymbol{required} \boldsymbol{distance}\left( \boldsymbol{D}_{\boldsymbol{ini}}\boldsymbol{,}\boldsymbol{N}_{\boldsymbol{tot}\boldsymbol{al}}\boldsymbol{,}\boldsymbol{N}_{\boldsymbol{c}\boldsymbol{ur}} \right)$  21 while $\left\vert\boldsymbol{P}_{\boldsymbol{new}} \right\vert\boldsymbol{<n}$ do  22 $\left( \boldsymbol{C}_{\boldsymbol{p}}\boldsymbol{,}\boldsymbol{C}_{\boldsymbol{np}} \right)\boldsymbol{\leftarrow}$categorize-individuals$\left( \boldsymbol{P}_{\boldsymbol{new}}\boldsymbol{,C,D} \right)$  23 if $\boldsymbol{C}_{\boldsymbol{np}}\boldsymbol{\neq\emptyset}$ then  24 $\boldsymbol{NDS\leftarrow}$non-dominated-set$\left( \boldsymbol{C}_{\boldsymbol{np}} \right)$  25 $\boldsymbol{T\leftarrow}$random-sampling$\left( \boldsymbol{NDS} \right)$  26 else  27 $\boldsymbol{T\leftarrow}$farthest$\left( \boldsymbol{C}_{\boldsymbol{p}}\boldsymbol{,NP} \right)$  28 end  29 $\boldsymbol{P}_{\boldsymbol{new}}\boldsymbol{\leftarrow}$ $\boldsymbol{P}_{\boldsymbol{new}}\boldsymbol{\cup}\left\{ \boldsymbol{T} \right\}$  30 $\boldsymbol{C\leftarrow C\backslash}\left\{ \boldsymbol{T} \right\}$  31 end  32 return $\boldsymbol{P}_{\boldsymbol{new}}$  33 end if  34 $\boldsymbol{gen\leftarrow gen+1}$  35 end while  36 return $\boldsymbol{P}_{\boldsymbol{maxGen}_{\boldsymbol{1}}}$  37 select a set of diverse individuals $\tilde{\boldsymbol{R}}$ from $\boldsymbol{P}_{\boldsymbol{maxGen}_{\boldsymbol{1}}}$  38 set the selected feature set $\boldsymbol{F\leftarrow\phi}$  39 for each $\boldsymbol{f=1 to}\left\vert\boldsymbol{F} \right\vert$ do  40 $\boldsymbol{vote(f)\leftarrow0}$  41 for each $\tilde{\boldsymbol{r}}\boldsymbol{\in R}$ do  42 calculate the contribution $\boldsymbol{Co}\boldsymbol{n}\left( \boldsymbol{f,}\tilde{\boldsymbol{r}} \right)$ by Eq (4)  43 calculate the voting weight $\boldsymbol{w}\boldsymbol{(}\tilde{\boldsymbol{r}}\boldsymbol{)}$ by Eq (5)-(7)  44 if $\boldsymbol{Co}\boldsymbol{n}\left( \boldsymbol{f,}\tilde{\boldsymbol{r}} \right)\boldsymbol{>0}$ then  45 $\mathbf{vote}\left( \mathbf{f} \right)\boldsymbol{\leftarrow vote}\left( \mathbf{f} \right)\mathbf{+}\mathbf{w}\mathbf{(}\tilde{\mathbf{r}}\mathbf{)}$  46 end  47 end  48 if $\boldsymbol{vote}\left( \boldsymbol{f} \right)\boldsymbol{\geq(}\sum_{\tilde{\boldsymbol{r}}\boldsymbol{=1}}^{\left\vert\boldsymbol{R} \right\vert} \boldsymbol{w}\boldsymbol{(}\tilde{\boldsymbol{r}}\boldsymbol{))/2}$ then  49 $\boldsymbol{F\leftarrow F\cup f}$  50 end  51 end  52 return $\boldsymbol{F}$  53 initialize the population ($\boldsymbol{P}_{\boldsymbol{maxGen}_{\boldsymbol{1}}}$) initialize with individual adaptation  54 evaluate the individuals in the population  55 for all $\boldsymbol{r}_{\boldsymbol{i}}\boldsymbol{\in}\boldsymbol{P}_{\boldsymbol{g}}$ do  56 evaluate $\boldsymbol{f}\left( \boldsymbol{r}_{\boldsymbol{i}} \right)$ by applying to each scenario $\boldsymbol{s}_{\boldsymbol{i}}\boldsymbol{\in S}$  57 if $\boldsymbol{f}\left( \boldsymbol{r}_{\boldsymbol{i}} \right)\boldsymbol{< f}\left( \boldsymbol{r}_{\boldsymbol{best}} \right)$ then  58 $\boldsymbol{r}_{\boldsymbol{best}}\boldsymbol{\leftarrow}\boldsymbol{r}_{\boldsymbol{i}}$  59 end if  60 end for  61 if $\boldsymbol{gen<maxGen-1}$ then  62 parent selection: select parents to generate offspring  63 evolution: generate a new population by crossover and mutation ($\boldsymbol{F}$)  64 end if  65 $\boldsymbol{gen\leftarrow gen+1}$  66 end while  67 return $\boldsymbol{r}_{\boldsymbol{best}}$ |
